# Supplementary material for: A structure-based epitope tagging approach identifies vulnerable sites on the malarial P36-P52 protein complex for antibody-mediated neutralization of Plasmodium sporozoites
Source: PLoS Pathog. 2026 Jul 8;22(7):e1014418. doi: 10.1371/journal.ppat.1014418 (PMC13372241; doi:10.1371/journal.ppat.1014418)
Supplement: S1 Table — (PDF) [file ppat.1014418.s001.pdf]

Table S2. Details on protein sequences used for structure modeling, recombinant protein production and genetic tagging.

	Construct	Sequence	Signal peptide	Tags	Cleavage site	MM (kDa)
**Recombinant protein production**	PfP52-P36	N45-D438-(GGGGS)$_4$-K81-S379	GP64	N-terminal 8xHis	TEV	84.2
	PfP52	N45-D438	GP64	N-terminal 8xHis	TEV	46.0
**Tagged constructs transgenic parasites**	P36-FlagC	M1-D352-(3xFlag)	PbP36	C-terminal 3xFlag		43.6
	FlagN-P36	M1-L69-(3xFlag)-K70-D352	PbP36	N-terminal 3xFlag		43.4
	P52-V5C	M1-R433-(V5 tag)-P434-W472	PbP52	C-terminal V5		56.8
	FlagN-P52	M1-T26-(3xFlag)-N27-W472	PbP52	N-terminal 3xFlag		57.8
	B9-FlagC	M1-N772-(3xFlag)-D773-L852	PbB9	C-terminal 3xFlag		103.0
	FlagN-B9	M1-D31-(3xFlag)-G32-L852	PbB9	N-terminal 3xFlag		103.2
**PbPfP52P36B9 constructs transgenic parasites**	PbPfP52	PbP52 M1-F22 / PfP52 S44-740 / PbP52 I319-W472	PbP52			55.0
	PbPfP36	PbP36 M1-S68 / PfP36 V80-S379	PbP36			43.3
	PbPfB9	PbB9 M1-K35 / PfB9 F29-I833 / PbB9 L776-L852	PbB9			108.9
**AF predictions P36-P52 heterodimers**	PfP36-P52	P67-S379 – S44-S320				
	PbP36-P52	L69-D352 – S23-N307				
	PvP36-P52	K61-S344 – C21-N297				
	PyP36-P52	N72-S356 – H34-S297				
**AF prediction P36-P36 and P52-P52 homodimers**	PfP36-PfP36	P67-S379 – P67-S379				
	PfP52-PfP52	S44-S320 – S44-S320				
	PbP36-PbP36	L69-D352 – L69-D352				
	PbP52-PbP52	S23-N307 – S23-N307				
	PvP36-PvP36	K61-S344 – K61-S344				
	PvP52-PvP52	H32-N297 – H32-N297				
	PyP36-PyP36	N72-S356 – N72-S297				
	PyP52-PyP52	H34-S297 – H34-S297				
**AF predictions P36 and P52 monomers**	PfP36	L78-S379				
	PfP52	S52-N328				
	PbP36	A67-D352				
	PbP52	T31-N307				
	PvP36	L58-V352				
	PvP52	S29-G305				
	PyP36	A71-S356				
	PyP52	T31-N307				
