# Supplementary material for: A structure-based epitope tagging approach identifies vulnerable sites on the malarial P36-P52 protein complex for antibody-mediated neutralization of Plasmodium sporozoites
Source: PLoS Pathog. 2026 Jul 8;22(7):e1014418. doi: 10.1371/journal.ppat.1014418 (PMC13372241; doi:10.1371/journal.ppat.1014418)
Supplement: S2 Table — (PDF) [file ppat.1014418.s002.pdf]

Table S2. SAXS data collection and scattering-derived parameters for *P. falciparum* P52-P36.

**Data collection parameters**	
Beam line	SWING (PROXIMA)
Wavelength (Å)	0.99
q range ($Å^{-1}$)*	0.0005 - 0.5543
Concentration (mg ml^{-1}) (mode)	4.2 (SEC-SAXS)
Buffer conditions	20 mM HEPES, 200 mM NaCl, 3% glycerol, pH 8.0
Temperature (°C)	20
**Structural parameters**§	
I(0) (cm^{-1}) [from Guinier]	0.85
R_g (Å) [from Guinier]	41.18
I(0) (cm^{-1}) [from *p(r)*]	0.87
R_g (Å) [from *p(r)*]	45.90
E.R.	2.06
D_{max} (Å)	~210
Porod volume estimate, V_p ($Å^3$)	179,887
Porod exponent	3.0
**Molecular mass determination**	
MM (kDa) [from *SAXSMoW* on final merged curve]	83.1 (q = 0.20 $Å^{-1}$)
MM (kDa) [from Q_R on final merged curve]	82.9 (q = 0.15 $Å^{-1}$)
MM (kDa) [from V_p/1.7]	105.8
Calculated MM from sequence (kDa)	84.4
**Ensemble modeling**	
Conformer generation and selection	BILBOMD
Protein regions selected to be rigid/fixed	19-289, 432-730
**Software employed**	
Data processing and analysis	ATSAS, BioXTAS RAW
**SASBDB**	
**Entry**	SASDY36

Abbreviations: *I(0)*, extrapolated scattering intensity at zero angle; R_g, radius of gyration calculated using either Guinier approximation (from Guinier) or the indirect Fourier transform package GNOM [from *p(r)*]; *MM*, molecular mass; D_{max}, maximal particle dimension; V_p, Porod volume; E.R., elongation ratio

*Momentum transfer $|q| = 4\pi\sin(\theta)/\lambda$
