# Supplementary figures and images for: A structure-based epitope tagging approach identifies vulnerable sites on the malarial P36-P52 protein complex for antibody-mediated neutralization of Plasmodium sporozoites
Source: PLoS Pathog. 2026 Jul 8;22(7):e1014418. doi: 10.1371/journal.ppat.1014418 (PMC13372241; doi:10.1371/journal.ppat.1014418)

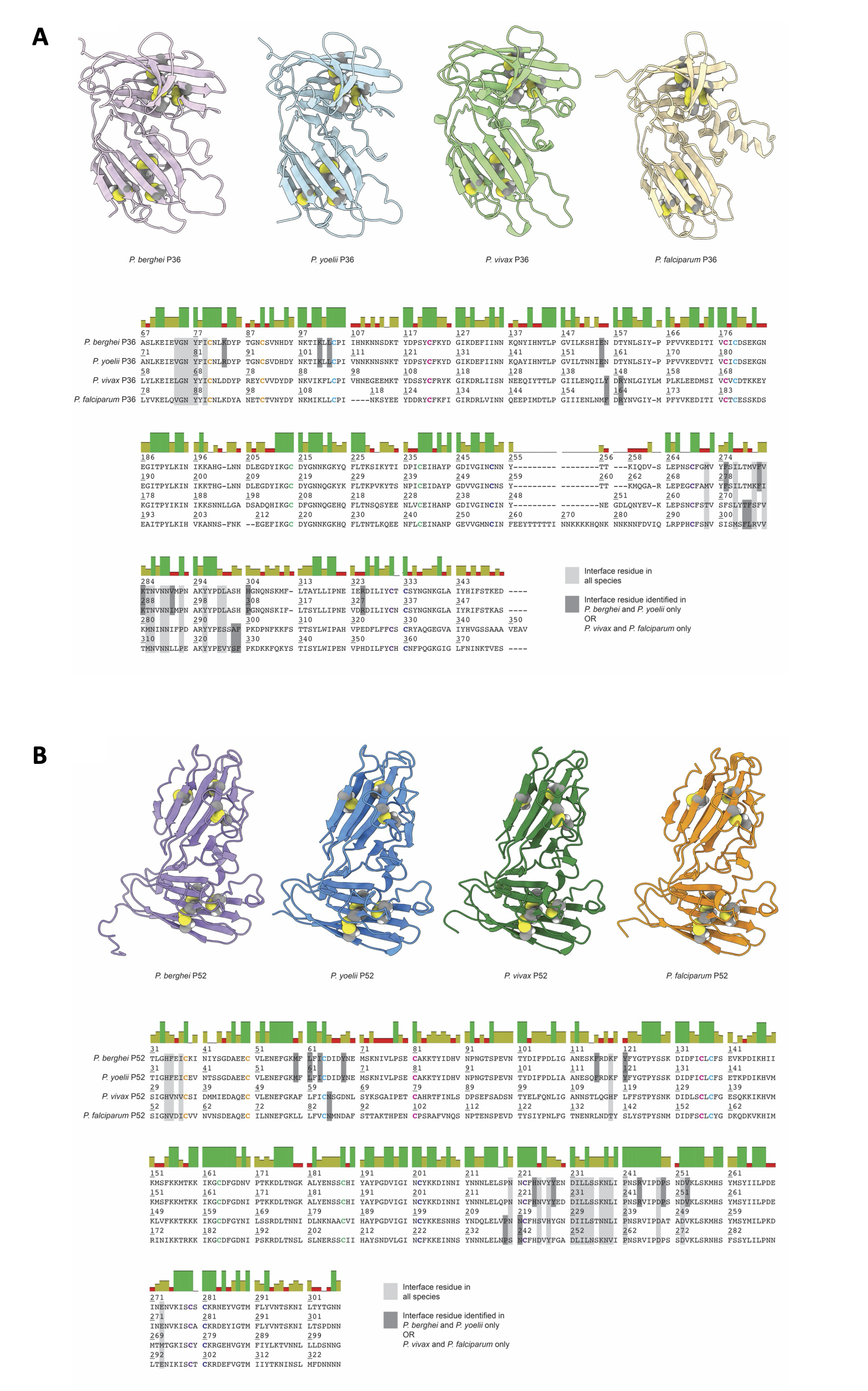

Supplement: S1 Fig — A. The top section shows cartoon representations of P36 AlphaFold models color-coded as in Fig 2. Disulfides are shown as spheres and colored by heteroatom. The bottom section displays a multiple sequence alignment (MSA) of P36 sequences from P. berghei, P. yoelii, P. vivax, and P. falciparum. Cysteines involved in the formation of an intramolecular disulfide are indicated in bold. Interface residues in all species are indicated by the light grey areas, whereas those only found in i) both P. berghei and P. yoelii or ii) both P. vivax and P. falciparum are highlighted by dark grey areas. The colored bars above the MSA represent the percentage of sequence identity: green (100%), green-brown (between 30% and 100%), and red (below 30%). B. Same analysis for P52. (TIFF) [file ppat.1014418.s005.tiff]

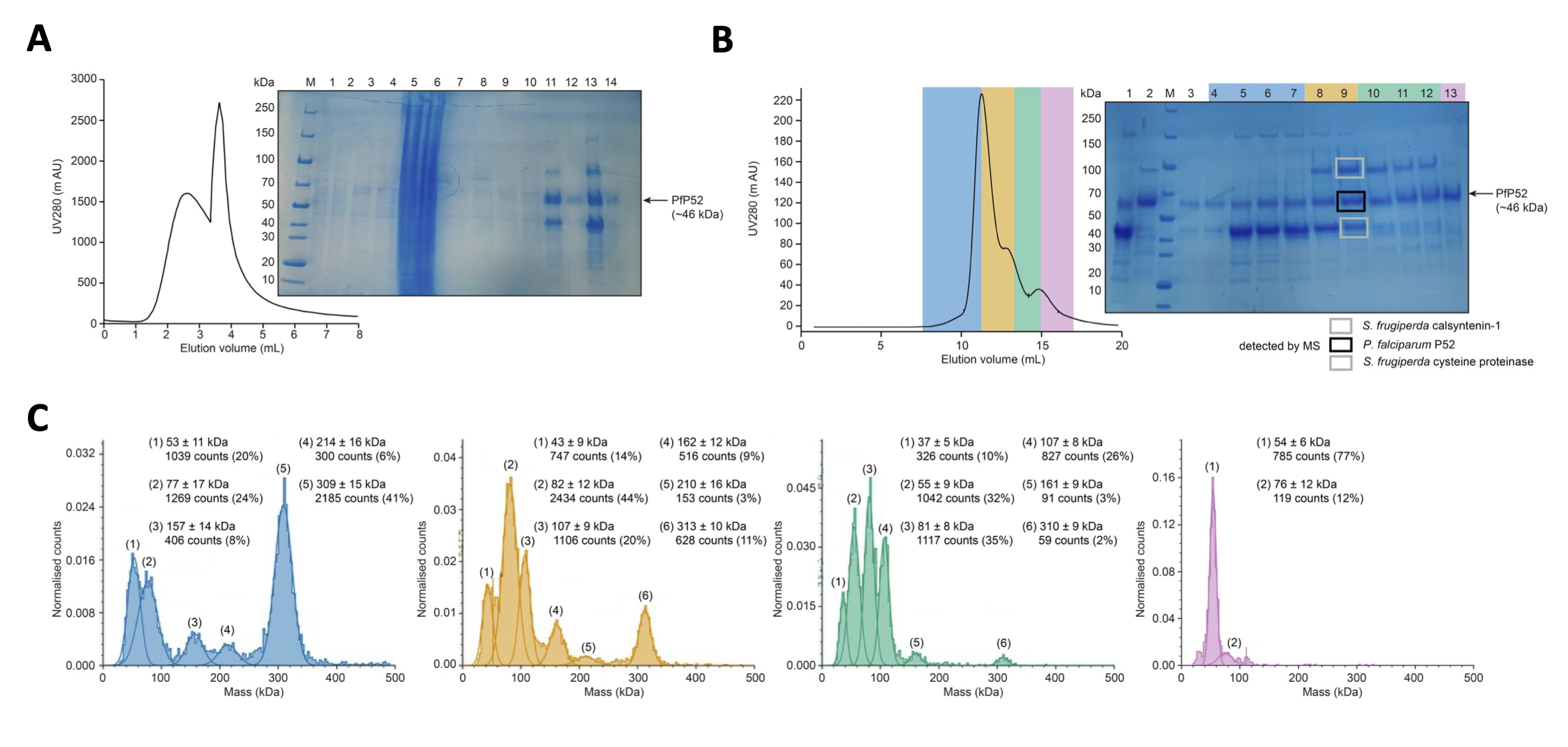

Supplement: S2 Fig — A. IMAC elution profile after having loaded the insect cell culture supernatant onto the column (Complete His-tag 1 mL, Roche). The elution was performed in a single step with 100% elution buffer and was paused after ~3 mL for ~5 minutes to incubate the column with the elution buffer to accelerate the process, as evidenced by the sudden increase in UV280 absorbance when elution is resumed. The inset shows an SDS-PAGE analysis of various analyzed fractions. Two IMACs were performed with samples from two cell cultures, grown and transfected in parallel. Lane 1, whole cell culture 1 before harvesting. Lane 2, whole cell culture 2 before harvesting. Lane 3, supernatant of centrifuged cell culture 1. Lane 4, supernatant of centrifuged cell culture 2. Lane 5, pellet of cell culture 1. Lane 6, pellet of cell culture 2. Lane 7, filtered supernatant of cell culture 1 loaded onto the column. Lane 8, filtered supernatant of cell culture 2 loaded onto the column. Lane 9, flow through of culture 1 after loading sample. Lane 10, flow through of culture 2 after loading sample. Lane 11, pooled elution fractions of IMAC 1. Lane 12, pre-elution fraction of IMAC 1. Lane 13, pooled elution fractions of IMAC 2. Lane 14, pre-elution fraction of IMAC 2. Lane M, PageRuler Unstained Broad Range Protein Ladder. The black arrow indicates the expected molecular mass for recombinant P. falciparum P52 (PfP52, ~ 46 kDa). B. SEC elution profile on pooled fractions after IMAC (fractions 2A and 2B). The volume of the pooled fractions was ~ 12 mL, two consecutive SEC runs were performed (Superdex 200 10/30 increase, Cytiva). The inset shows an SDS-PAGE analysis of various analyzed fractions from one of the two identical runs. Lanes 1 and 2, fractions from the other SEC run. Lane 3, fraction 1. Lanes 4–7, fractions 2 until 5, which fall under the area highlighted in blue. Lanes 8 and 9, fractions 6 and 7, which fall under the area highlighted in orange. Lanes 10–12, fractions 8 until 10, which fall un [file ppat.1014418.s006.tiff]

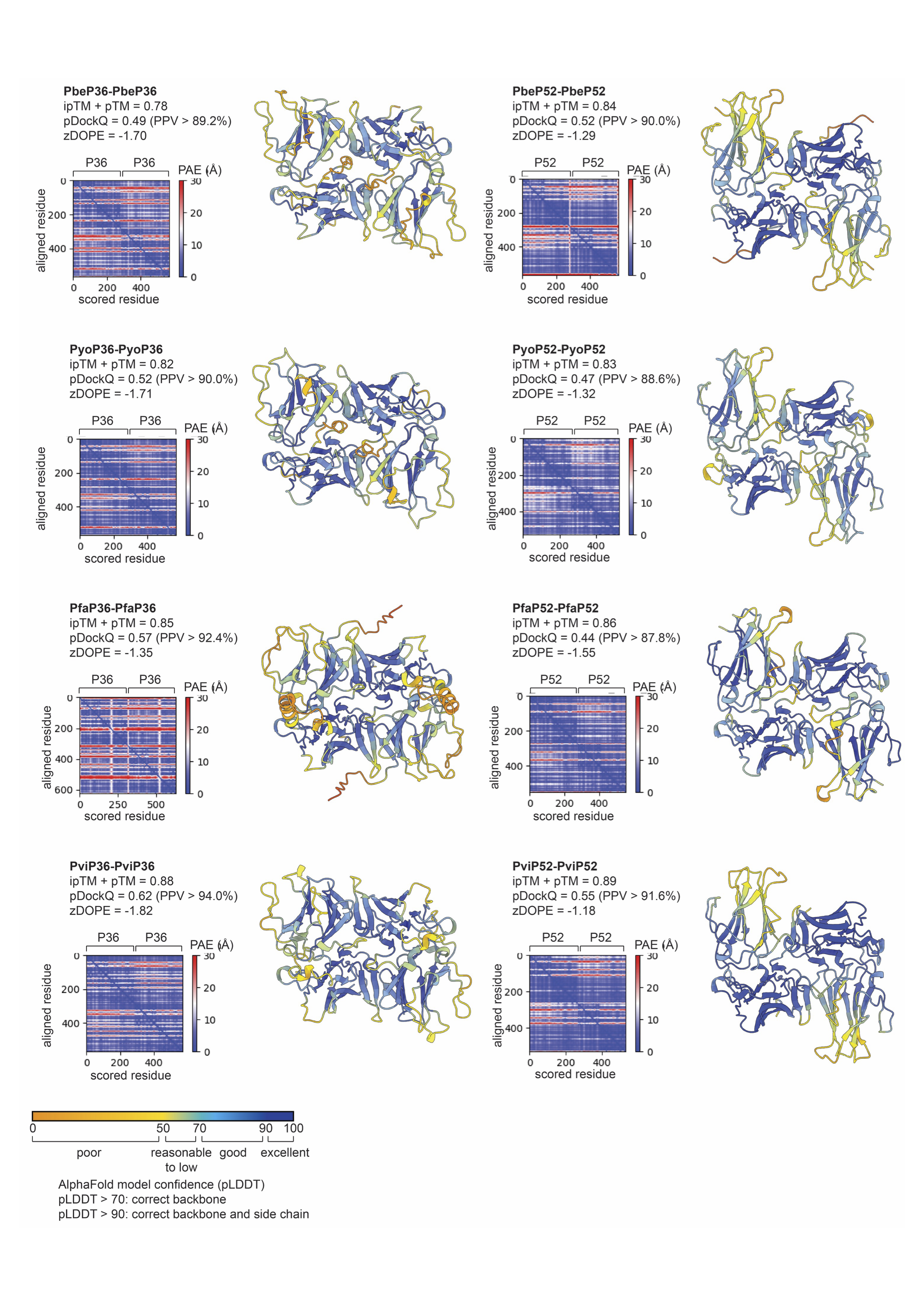

Supplement: S3 Fig — The models are displayed as cartoon representations and are colored according to the predicted local distance difference test (pLDDT) score, which reflects (local) model quality as indicated by the legend at the bottom. For all structures, the predicted aligned error (PAE), the normalized discrete optimized protein energy (zDOPE), the pDockQ and AlphaFold-Multimer model confidence (0.8*ipTM + 0.2*pTM) are also shown. (TIFF) [file ppat.1014418.s007.tiff]

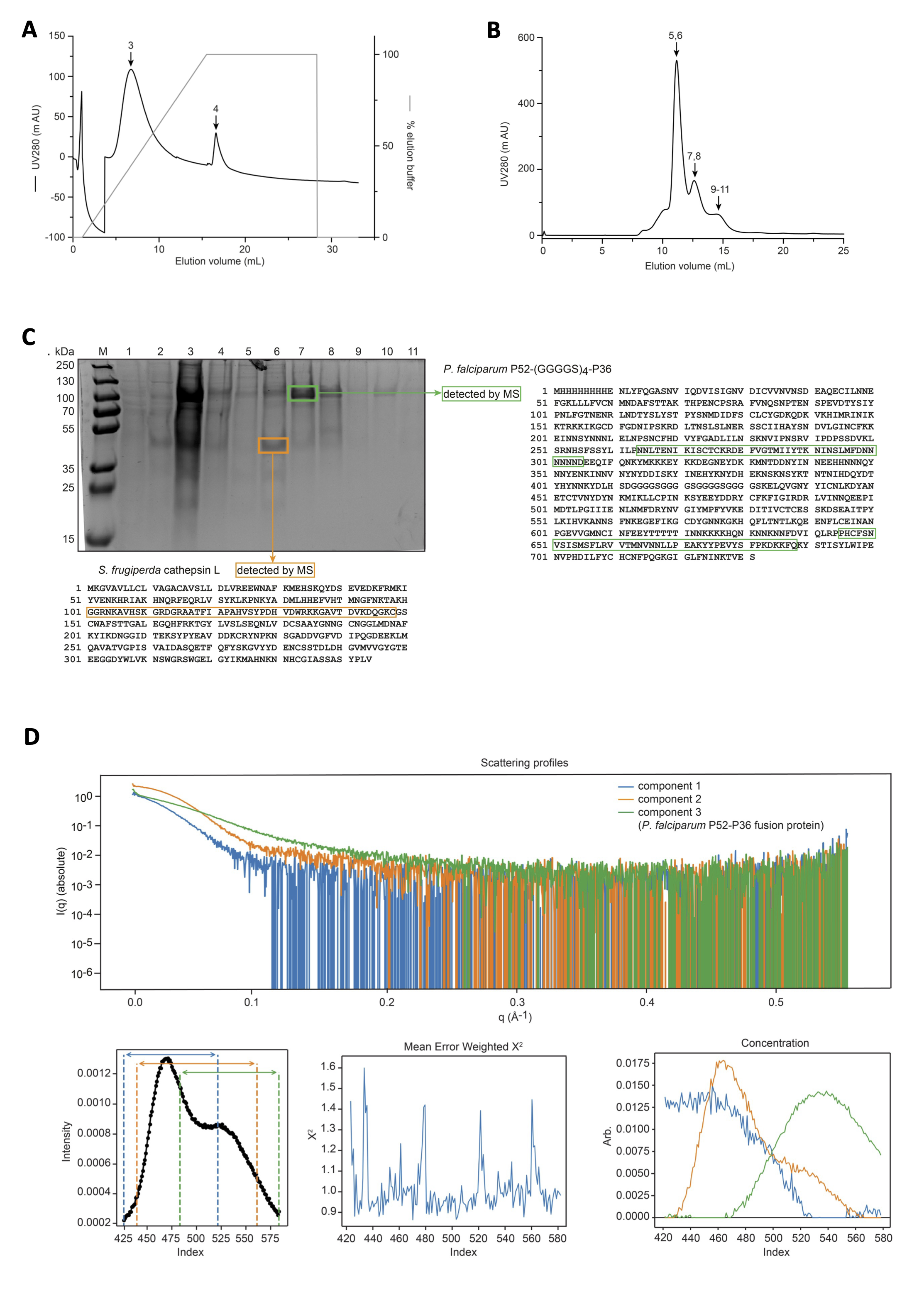

Supplement: S4 Fig — A. IMAC elution profile after having loaded the insect cell culture supernatant onto the column (HisTrap HP 1 mL, Cytiva). The elution was performed through an elution gradient. B. SEC elution profile on pooled fractions after IMAC (fractions 2–6). C. SDS-PAGE analysis of the fractions collected during SEC and IMAC. Lane 1, filtered supernatant of cell culture 1 loaded onto the column. Lane 2, wash fraction prior to elution. Lane 3, IMAC elution peak. Lane 4, IMAC elution peak. Lanes 5–11, SEC elution fractions. Lane M, PageRuler Plus PreStained Protein Ladder. The colored boxes indicate the excised gel bands that were analyzed via MS to confirm the identity of the P. falciparum P52-P36 fusion construct (~83 kDa) and the insect cell contaminant (~38 kDa). D. Screenshot of the SAXS data processing employing evolving factor analysis to deconvolute the elution peaks obtained during SEC-SAXS to obtain the scattering curve for the P. falciparum P52-P36 fusion construct as presented in Fig 3. The evolving factor analysis identifies three scattering components, of which the third component (colored green) corresponds to the P. falciparum P52-P36 fusion construct. (TIFF) [file ppat.1014418.s008.tiff]

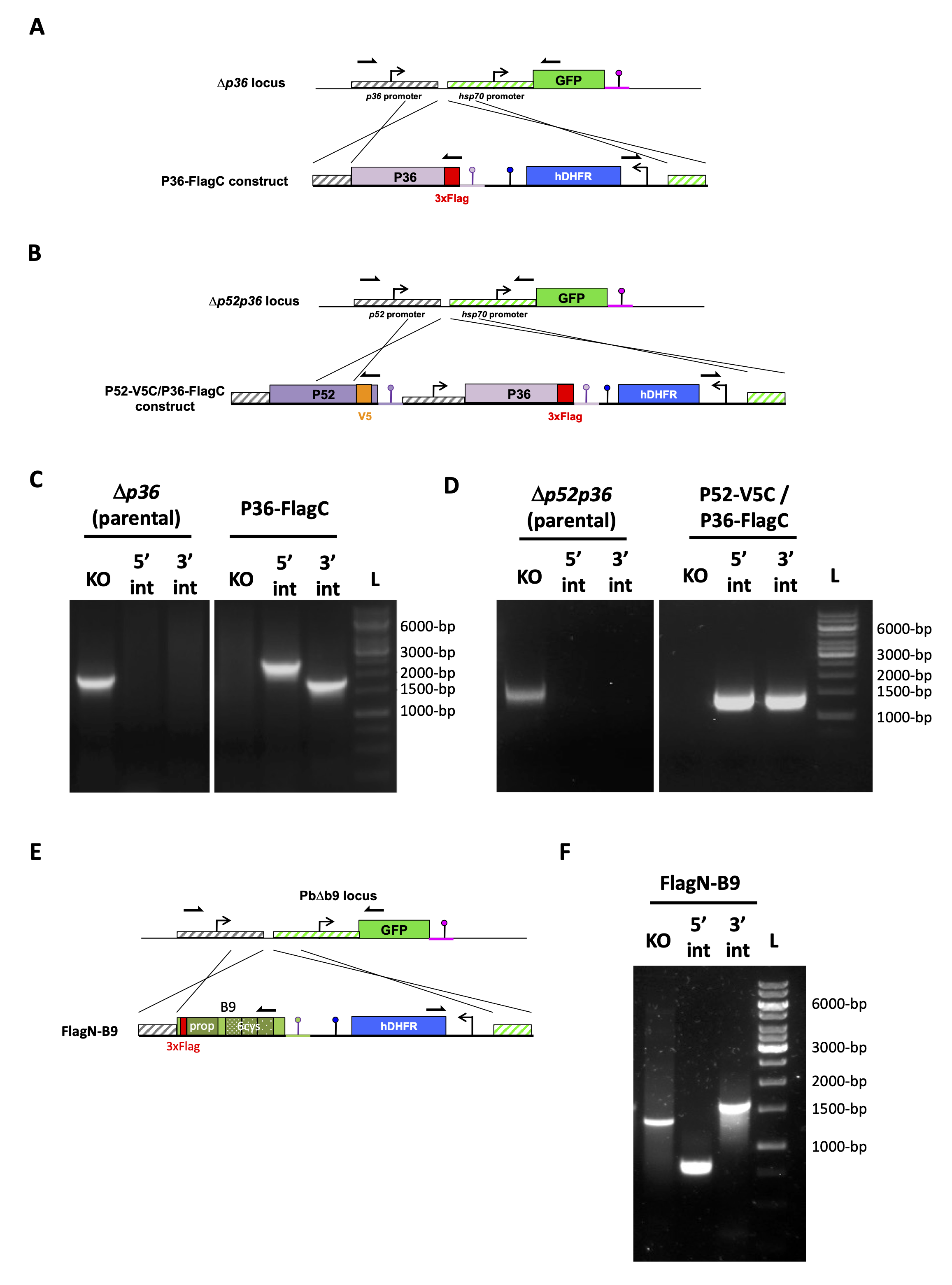

Supplement: S5 Fig — Genetic strategy to generate the P36-FlagC parasites, based on genetic complementation of Δp36 with a construct harboring a P36 coding sequence with a C-terminal 3xFlag in addition to a hDHFR pyrimethamine resistance cassette. B. Genetic strategy to generate the P52-V5C/P36-FlagC parasites, based on genetic complementation of Δp52p36 with a construct harboring a P52 coding sequence with a C-terminal V5 epitope, a P36 coding sequence with a C-terminal 3xFlag, and the hDHFR pyrimethamine resistance cassette. C-D. Genotyping of P36-FlagC (C) and P52-V5C/P36-FlagC (D) parasites by PCR using primers combinations specific for the parental genome (WT) or for the 5’ and 3’ recombination events. E. Genetic strategy to generate the FlagN-B9 parasites, based on genetic complementation of GFP-expressing Δb9 with a construct harboring a B9 coding sequence with a N-terminal 3xFlag epitope in addition to a hDHFR pyrimethamine resistance cassette. F. Genotyping of FlagN-B9 parasites by PCR using primers combinations specific for the parental genome (WT) or for the 5’ and 3’ recombination events. (TIFF) [file ppat.1014418.s009.tiff]

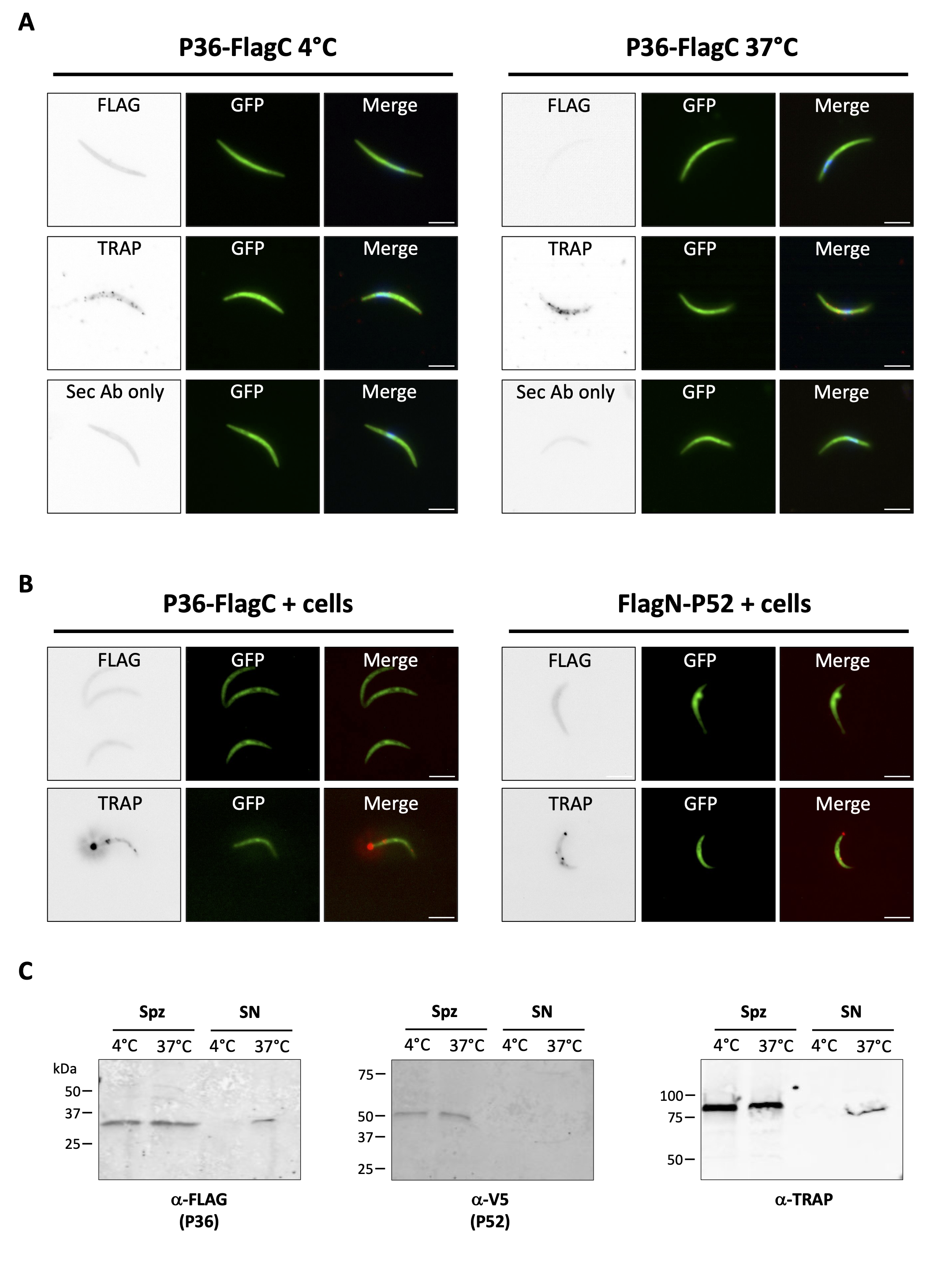

Supplement: S6 Fig — A. Immunofluorescence analysis of control versus activated P36-FlagC SPZs after staining with anti-Flag or anti-TRAP antibodies (red) and Hoechst 33342 (blue). Microneme secretion was stimulated by incubation for 15 min at 37˚C in a buffer containing 1% BSA and 1% ethanol. In the control condition, parasites were kept at 4°C. Parasites were fixed without permeabilization. Scale bar, 5 μm. B. Immunofluorescence analysis of P36-FlagC and FlagN-P52 SPZs after staining with anti-Flag or anti-TRAP antibodies (red). SPZs were incubated with HepG2 cells to stimulate microneme secretion, and fixed without permeabilization. Scale bar, 5 μm. C. Microneme secretion assay using P52-V5C/P36-FlagC SPZ (25 x 104 or equivalent per lane). Microneme secretion was stimulated by incubation for 15 min at 37°C in the presence of BSA and ethanol. In the control condition, parasites were kept at 4°C. Following activation, samples were fractionated by centrifugation in SPZ pellets (spz) and supernatants containing secreted proteins (SN), and analyzed by western blot using antibodies against Flag, V5 or TRAP. (TIFF) [file ppat.1014418.s010.tiff]

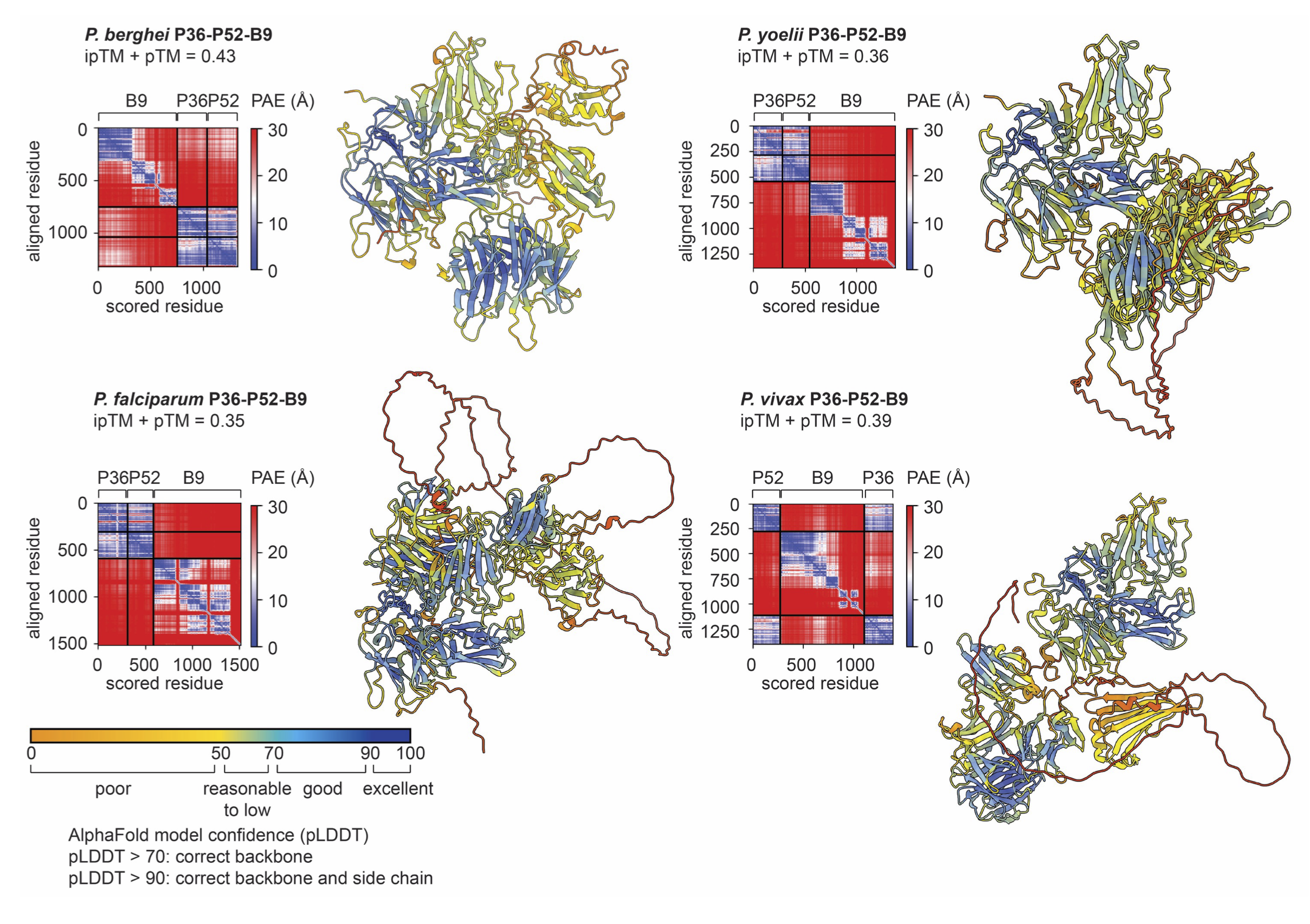

Supplement: S7 Fig — The heterotrimer models of P36-P52-B9 in P. berghei, P. yoelii, P. vivax, and P. falciparum are displayed as cartoon representations and are colored according to the predicted local distance difference test (pLDDT) score, which reflects (local) model quality as indicated by the legend at the bottom. For all structures, the predicted aligned error (PAE) and AlphaFold-Multimer model confidence (0.8*ipTM + 0.2*pTM) are also shown. (TIFF) [file ppat.1014418.s011.tiff]

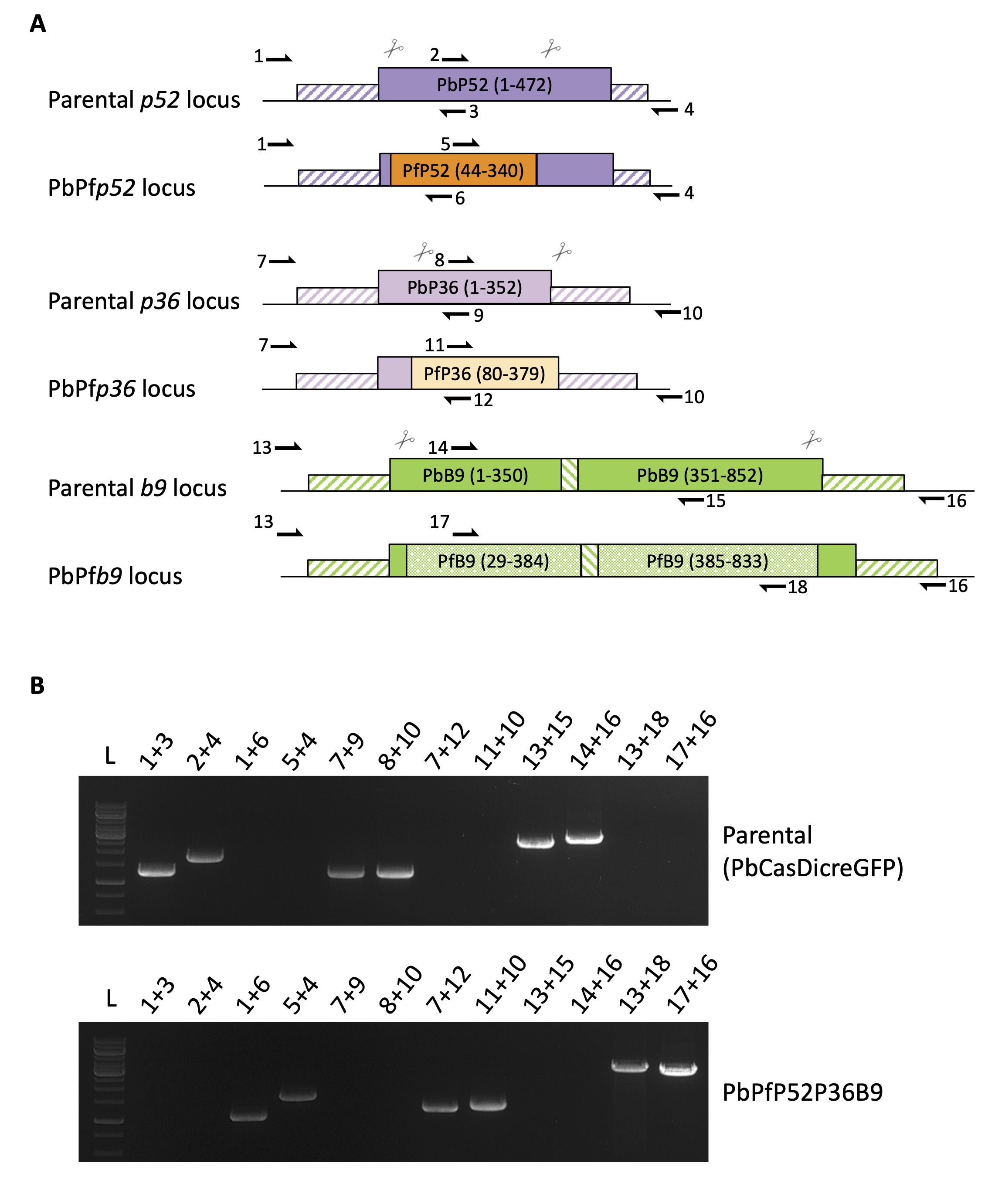

Supplement: S8 Fig — A. Strategy to replace PbP52, PbP36 and PbB9 in a PbCasDiCreGFP parasite line using CRISPR to successively generate falciparumized PbPfP52, PbPfP52P36 and PbPfP52P36B9 lines. The PbCasDiCreGFP parasites were co-transfected with a linearized DNA repair construct synthetically designed with a plasmid encoding gene specific sgRNA guides and a pyrimethamine-resistance cassette (hDHFR). B. PCR analysis of the genomic DNA obtained from the parental PbCasDiCreGFP line and the recombinant line PbPfP52P36B9. Confirmation of the expected recombination events was achieved with primer combinations specific for P. berghei or P. falciparum gene sequences. (TIFF) [file ppat.1014418.s012.tiff]
