# Supplementary material for: A structure-based epitope tagging approach identifies vulnerable sites on the malarial P36-P52 protein complex for antibody-mediated neutralization of Plasmodium sporozoites
Source: PLoS Pathog. 2026 Jul 8;22(7):e1014418. doi: 10.1371/journal.ppat.1014418 (PMC13372241; doi:10.1371/journal.ppat.1014418)
Supplement: S1 File — Uncropped gel and blot images. (PDF) [file ppat.1014418.s013.pdf]

Uncropped image for S2A Fig

L 1 2 3 4 5 6 7 8 9 10 11 12 13 14

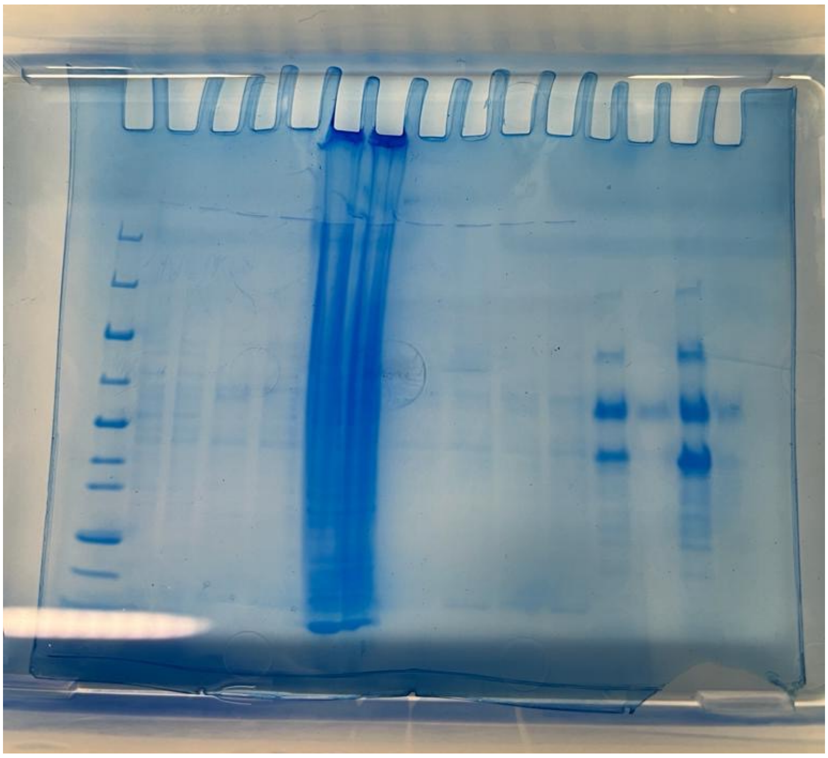

L = ladder
1 = Whole cell culture 1 before harvesting
2 = Whole cell culture 2 before harvesting
3 = Supernatant of centrifuged cell culture 1
4 = Supernatant of centrifuged cell culture 2
5 = Pellet of cell culture 1
6 = Pellet of cell culture 2
7 = Filtered supernatant of cell culture 1 loaded onto column
8 = Filtered supernatant of cell culture 2 loaded onto column
9 = Flow through of culture 1 after loading sample
10 = Flow through of culture 2 after loading sample
11 = Pooled elution fractions of IMAC 1
12 = Pre-elution fraction of IMAC 1
13 = Pooled elution fractions of IMAC 2
14 = Pre-elution fraction of IMAC 2

Uncropped image for S2B Fig

1 2 L 3 4 5 6 7 8 9 10 11 12 13 X

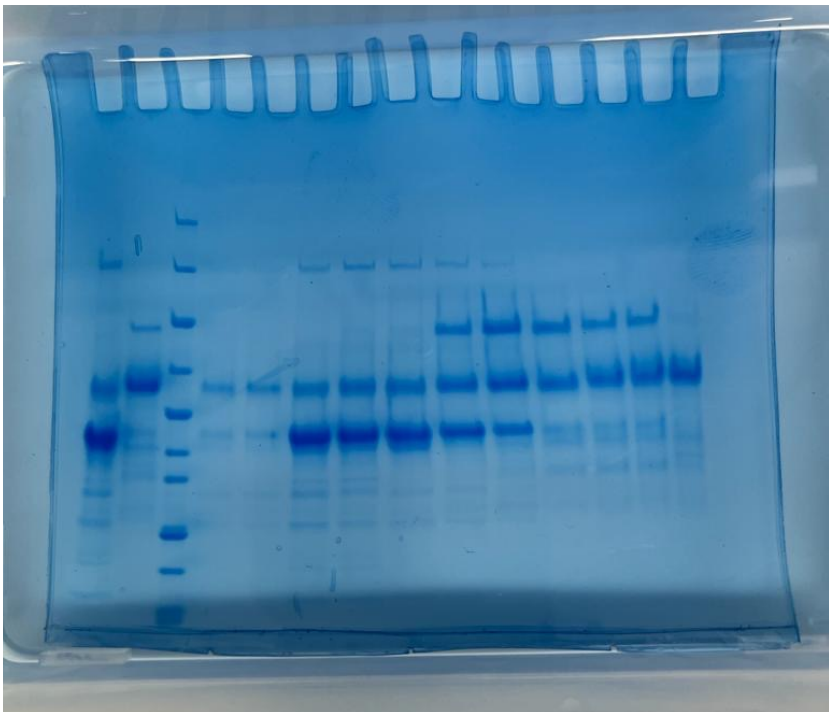

L = ladder
1 = Fraction from the other SEC run (2 runs in parallel)
2 = Fraction from the other SEC run (2 runs in parallel)
3 = SEC elution fraction 1
4 = SEC elution fraction 2
5 = SEC elution fraction 3
6 = SEC elution fraction 4
7 = SEC elution fraction 5
8 = SEC elution fraction 6
9 = SEC elution fraction 7
10 = SEC elution fraction 8
11 = SEC elution fraction 9
12 = SEC elution fraction 10
13 = SEC elution fraction 11
X = not included in Fig

Uncropped image for S4C Fig

L 1 2 3 4 5 6 7 8 9 10 11 X X X

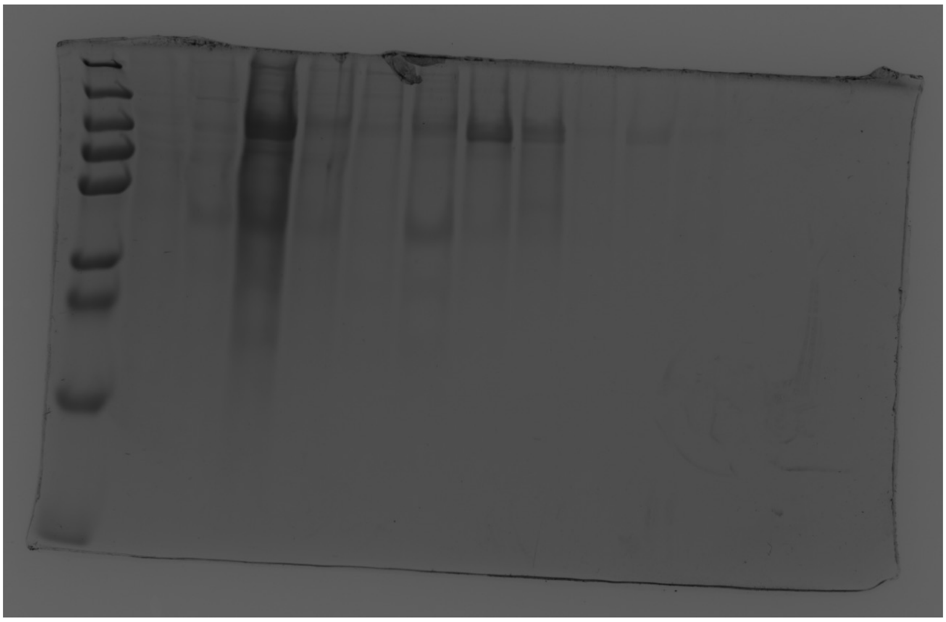

L = ladder
1 = Filtered supernatant of cell culture 1 loaded onto column
2 = Wash fraction prior to elution
3 = IMAC elution peak
4 = IMAC elution peak
5 = SEC elution fraction
6 = SEC elution fraction
7 = SEC elution fraction
8 = SEC elution fraction
9 = SEC elution fraction
10 = SEC elution fraction
11 = SEC elution fraction
X = not included in Fig

Uncropped image for S5C Fig

X X X 1 2 3 X X X 4 5 6 L

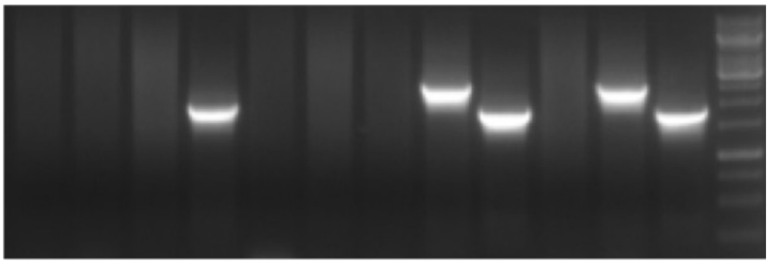

L = ladder
1 = Δ*p36* gDNA / KO primers
2 = Δ*p36* gDNA / 5’int primers
3 = Δ*p36* gDNA / 3’int primers
4 = P36-FlagC gDNA / KO primers
5 = P36-FlagC gDNA / 5’int primers
6 = P36-FlagC gDNA / 3’int primers
X = not included in figure

Uncropped image for S5D Fig

L 1 2 3 X X X L X X X 4 5 6 L

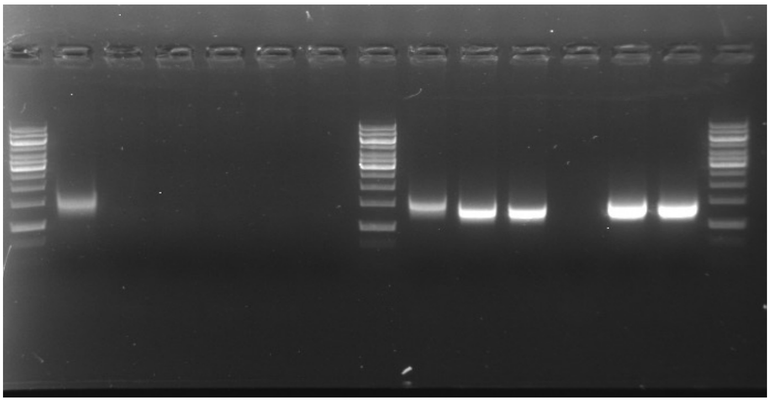

L = ladder
1 = Δ*p52p36* gDNA / KO primers
2 = Δ*p52p36* gDNA / 5’int primers
3 = Δ*p52p36* gDNA / 3’int primers
4 = P52-V5C/P36-FlagC gDNA / KO primers
5 = P52-V5C/P36-FlagC gDNA / 5’int primers
6 = P52-V5C/P36-FlagC gDNA / 3’int primers
X = not included in figure

Uncropped image for S5F Fig

X L X X X X X X X X X 1 2 3 L

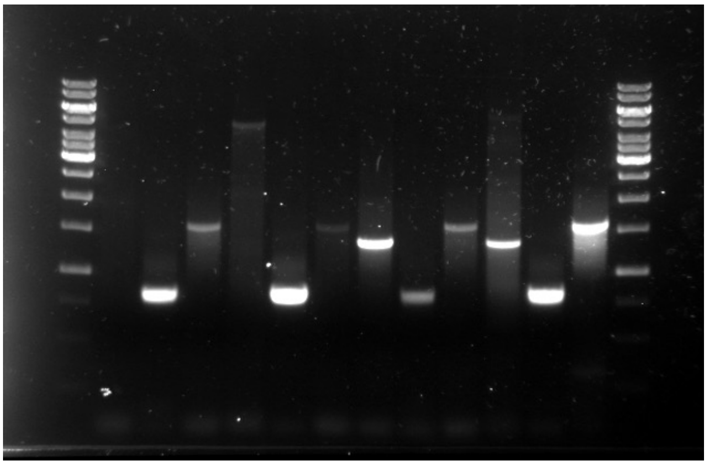

L = ladder
1 = FlagN-B9 gDNA/ KO primers
2 = FlagN-B9 gDNA / 5’int primers
3 = FlagN-B9 gDNA / 3’int primers
X = not included in figure

Uncropped image for S6C Fig (anti-Flag blot)

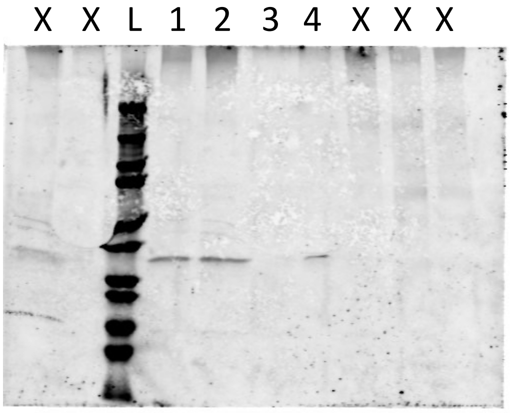

L = ladder
1 = 4°C spz pellet
2 = 37°C spz pellet
3 = 4° spz supernatant
4 = 37° C spz supernatant
X = not included in Fig

Uncropped image for S6C Fig (anti-V5 blot)

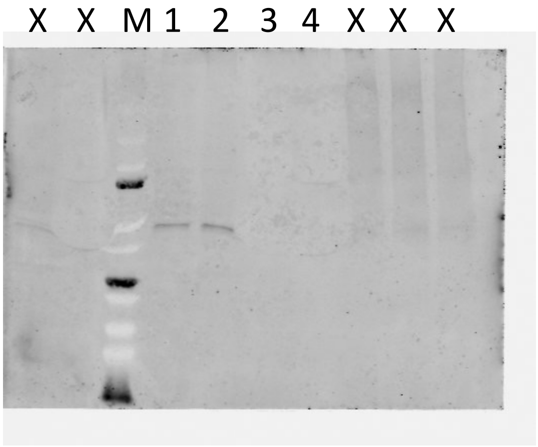

L = ladder
1 = 4°C spz pellet
2 = 37°C spz pellet
3 = 4° spz supernatant
4 = 37° C spz supernatant
X = not included in Fig

Uncropped image for S6C Fig (anti-TRAP blot)

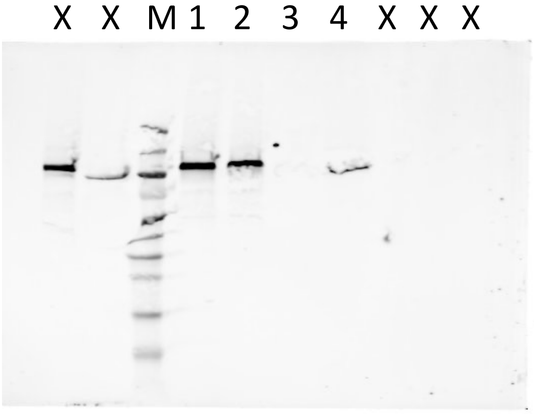

L = ladder
1 = 4°C spz pellet
2 = 37°C spz pellet
3 = 4° spz supernatant
4 = 37° C spz supernatant
X = not included in Fig

Uncropped image for S8B Fig (parental)

L 1 2 3 4 5 6 7 8 9 10 11 12

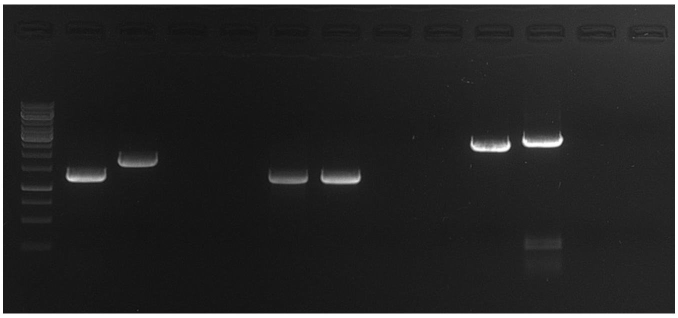

Uncropped image for S8B Fig (PbPfP52P36B9)

L 1 2 3 4 5 6 7 8 9 10 11 12

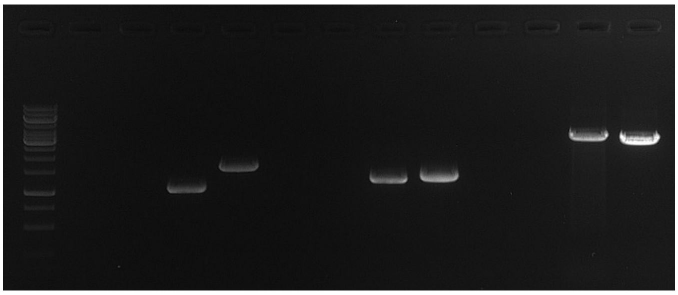

L = ladder
1 = primers 1+3
2 = primers 2+4
3 = primers 1+6
4 = primers 5+4
5 = primers 7+9
6 = primers 8+10
7 = primers 7+12
8 = primers 11+10
9 = primers 13+15
10 = primers 14+16
11 = primers 13+18
12 = primers 17+16
