# Supplementary material for: A structure-based epitope tagging approach identifies vulnerable sites on the malarial P36-P52 protein complex for antibody-mediated neutralization of Plasmodium sporozoites
Source: PLoS Pathog. 2026 Jul 8;22(7):e1014418. doi: 10.1371/journal.ppat.1014418 (PMC13372241; doi:10.1371/journal.ppat.1014418)
Supplement: Attachment — Submitted filename: Das Boeykens et al_Response_to_reviewers.pdf [file ppat.1014418.s015.pdf]

Reply to the reviewers (PPATHOGENS-D-26-00836)
A structure-based epitope tagging approach identifies vulnerable sites on the malarial P36-P52 protein complex for antibody-mediated neutralization of *Plasmodium* sporozoites

Samhita Das, Lien Boeykens, Manon Loubens, Carine Marinarch, Sylvie Briquet, Line De Vocht, Isabel Pintelon, Jean-Pierre Timmermans, Yann G.-J. Sterckx, Olivier Silvie

First and foremost, we would like to thank the reviewers and editorial staff for their thorough review of our manuscript and for their positive and useful comments. As detailed below in our point-by-point response, we have now addressed all the comments (our responses appear in blue).

ADDITIONAL EDITOR COMMENTS

In addition to addressing the reviewer comments in full, please pay particular attention to the following points when revising the manuscript:

First, please moderate the interpretation of the anti-tag antibody experiments. The current data show that antibodies against engineered epitope tags can inhibit invasion, but they do not yet establish that native P36/P52 epitopes are naturally immunogenic or that antibodies against native P36/P52 sequences would neutralize infection. Claims relating to vulnerable epitopes, vaccine relevance, or therapeutic antibody targeting should therefore be revised accordingly unless additional supporting data are provided.

Reply: Indeed, the epitope tagging neutralization approach shows that the P36-P52 complex is accessible to the action of neutralizing antibodies, but does not address whether P36/P52 contain immunogenic sequences nor that antibodies targeting native epitopes would inhibit infection. The same limitation stands for the experiments with B9. We have modified the text accordingly to make it clear that inhibition is mediated by anti-tag antibodies against engineered epitope tags, and we explicitly mention the limitation relative to native epitopes. Nevertheless, based on our data identifying domains in the P36-P52 heterodimer that are accessible to antibody neutralization, we believe that these antigens should be considered as potential new targets. We acknowledge that future studies will be required to determine whether native sequences are immunogenic and associated with protection against infection.

Second, please clarify and expand the description of all recombinant and parasite constructs used in the study, including the precise sequences, boundaries, tags, signal peptides, linkers, and relevant expression-design choices.

Reply: We agree with this suggestion and have generated an additional table in the 'Supporting Information' (**Table S1**) that contains a detailed overview of all constructs employed in this work.

Third, please temper the language around the SAXS and negative-stain EM data. The reviewers were concerned that these data are more appropriately described as being consistent with the predicted P36-P52 architecture rather than as definitively validating it, unless stronger experimental evidence is provided.

Reply: We fully agree with this comment and have adapted the text accordingly.

Fourth, please address the apparent discrepancy between the reported difficulty detecting P36/P52 on the sporozoite surface and the ability of anti-tag antibodies to inhibit invasion. The manuscript would benefit from a clearer discussion, and where possible, additional data, explaining when and how these epitopes are likely to become antibody-accessible.

Reply: As requested, we provide additional data (**new Fig S6**) to document that we could not detect P36 or P52 on the surface of activated sporozoites, using two different activation protocols (stimulation of microneme secretion by EtOH/BSA or in the presence of host cells). In both conditions TRAP was readily detected on the surface of non-permeabilized sporozoites (**new Fig S6A-B**). Importantly, P36 (but not P52) could be detected in the supernatant of

activated sporozoites (**new Fig S6C**) confirming that it is exposed extracellularly. We also show that anti-Flag antibodies do not inhibit cell traversal of sporozoites expressing Flag-tagged P36 or P52 (**new Fig 4G**). Our results are in line with previous work showing that P36 and P52 were not detected on the sporozoite surface (refs 30, 45, 46). Altogether, our results suggest that P36-P52 are only exposed briefly at the parasite surface and/or in very small amounts. We have modified the text in the discussion as follows:

"*Our data show that anti-Flag antibodies inhibit infection of host cells by tagged parasites but have no effect on sporozoite cell traversal activity, consistent with the exclusive role of P36 and P52 during productive invasion [16,31,34,41]. It is possible that, following microneme secretion, P36 and P52 are exposed only briefly at the parasite surface at the time of host cell invasion, and/or in very small amounts, below the detection limit. Transient surface exposure would still allow high affinity antibodies (such as the anti-tag monoclonal antibodies used in this study) to bind to the P36-P52 complex and block its function*."

Finally, please substantially reconsider the interpretation of the triple PfP36/PfP52/PfB9 substitution experiment. As currently presented, the failure of these parasites to invade HepG2 cells is difficult to interpret as evidence against a functional P36/P52/B9 complex. This phenotype could have several explanations, including altered expression, folding, trafficking, localization, species-specific incompatibility, or dependence on additional P. falciparum factors. Please either provide additional evidence supporting the expression, localization, and appropriate trafficking of the substituted proteins, or reframe this result more cautiously as a negative observation that does not by itself exclude a functional P36/P52/B9 complex.

<u>*Reply:*</u> we fully agree that both the AlphaFold models and the functional data with the triple PfP52P36B9 substituted parasites are negative results, and that we cannot rule out that these proteins assemble in a functional trimeric complex. We have modified the text accordingly to reconsider the interpretation of these results:

"*We also investigated the existence of a possible functional link between the P36-P52 complex and B9, since P36/P52 and B9 mutants display similar phenotypes [16,31–33]. Our data show that genetically engineered P. berghei SPZs carrying P. falciparum P36, P52 and B9 coding sequences are non-infective, strongly suggesting that additional factors are required for SPZ invasion of host cells. Alternatively, altered expression, trafficking or folding of P. falciparum proteins in P. berghei SPZs could also explain this phenotype. We also found that AlphaFold predictions of the P. falciparum, P. berghei, P. yoelii and P. vivax P36-P52-B9 complexes display very poor confidence scores. Hence, apart from the shared phenotype of gene deletion mutants, it remains unclear whether there is any functional or structural link between the P36-P52 heterodimer and B9. Further work is thus needed to elucidate the molecular function of these SPZ 6-Cys proteins.*"

JOURNAL REQUIREMENTS

If the reviewer comments include a recommendation to cite specific previously published works, please review and evaluate these publications to determine whether they are relevant and should be cited. There is no requirement to cite these works unless the editor has indicated otherwise.

1) Please ensure that the CRediT author contributions listed for every co-author are completed accurately and in full.
At this stage, the following Authors/Authors require contributions: Samhita Das, Lien Boeykens, Manon Loubens, Carine Marinach, Sylvie Briquet, Line De Vocht, Isabel Pintelon, Jean-Pierre Timmermans, Yann Sterckx, and Olivier Silvie. Please ensure that the full contributions of each author are acknowledged in the "Add/Edit/Remove Authors" section of our submission form.
The list of CRediT author contributions may be found here: https://journals.plos.org/plospathogens/s/authorship#loc-author-contributions

Reply: All CRediT author contributions have been accurately listed.

2) We ask that a manuscript source file is provided at Revision. Please upload your manuscript file as a .doc, .docx, .rtf or .tex. If you are providing a .tex file, please upload it under the item type u2018LaTeX Source Fileu2019 and leave your .pdf version as the item type u2018Manuscriptu2019.

Reply: A manuscript source file has been provided.

3) Please provide an Author Summary. This should appear in your manuscript between the Abstract (if applicable) and the Introduction, and should be 150-200 words long. The aim should be to make your findings accessible to a wide audience that includes both scientists and non-scientists. Sample summaries can be found on our website under Submission Guidelines:
https://journals.plos.org/plospathogens/s/submission-guidelines#loc-parts-of-a-submission

Reply: An Author Summary has been provided.

4) We noticed that you used the phrase 'data not shown' in the manuscript. We do not allow these references, as the PLOS data access policy requires that all data be either published with the manuscript or made available in a publicly accessible database. Please amend the supplementary material to include the referenced data or remove the references.

Reply: we have adapted the text accordingly and have included additional data (**new Fig S6**).

5) We do not publish any copyright or trademark symbols that usually accompany proprietary names, eg ©, ®, or TM (e.g. next to drug or reagent names). Therefore please remove all instances of trademark/copyright symbols throughout the text, including:
- TM on pages: 24, and 30.

Reply: We have adapted the text accordingly.

6) Please upload all main figures as separate Figure files in .tif or .eps format. For more information about how to convert and format your figure files please see our guidelines: https://journals.plos.org/plospathogens/s/figures

Reply: All figures have been uploaded as separate files in .tif format.

7) We notice that your supplementary Figures are included in the manuscript file. Please remove them and upload them with the file type 'Supporting Information'. Please ensure that each Supporting Information file has a legend listed in the manuscript after the references list.

Reply: The supplementary figures have now been uploaded as 'Supporting Information' files.

8) Some material included in your submission may be copyrighted. According to PLOS's copyright policy, authors who use figures or other material (e.g., graphics, clipart, maps) from another author or copyright holder must demonstrate or obtain permission to publish this material under the Creative Commons Attribution 4.0 International (CC BY 4.0) License used by PLOS journals. Please closely review the details of PLOS's copyright requirements here: PLOS Licenses and Copyright. If you need to request permissions from a copyright holder, you may use PLOS's Copyright Content Permission form.

Please respond directly to this email and provide any known details concerning your material's license terms and permissions required for reuse, even if you have not yet obtained copyright permissions or are unsure of your material's copyright compatibility. Once you have responded and addressed all other outstanding technical requirements, you may resubmit your manuscript within Editorial Manager.

Potential Copyright Issues:

i) Figure 4A. Please confirm whether you drew the images / clip-art within the figure panels by hand. If you did not draw the images, please provide (a) a link to the source of the images or icons and their license / terms of use; or (b) written permission from the copyright holder to publish the images or icons under our CC BY 4.0 license. Alternatively, you may replace the images with open source alternatives. See these open source resources you may use to replace images / clip-art:
- https://commons.wikimedia.org
- https://openclipart.org/.

Reply: **Figure 4A** has been generated using BioRender.com and we now explicitly mention this in the figure legend.

ii) The following Figures contain screenshots: S4, and S7A. We are not permitted to publish these under our CC-BY 4.0 license, websites are usually intellectual property and are copyrighted. This includes peripheral graphics of the web browser such as icons and button. We ask that you please remove or replace it.

<u>Reply:</u> **Figure S4D** was indeed generated through a screenshot, and we have regenerated all subfigures for this panel. **Figure S7A** was not obtained through a screenshot and we have therefore not adapted this panel.

9) Please amend your detailed Financial Disclosure statement. This is published with the article. It must therefore be completed in full sentences and contain the exact wording you wish to be published.
1) State what role the funders took in the study. If the funders had no role in your study, please state: "The funders had no role in study design, data collection and analysis, decision to publish, or preparation of the manuscript."
2) If any authors received a salary from any of your funders, please state which authors and which funders..
If you did not receive any funding for this study, please simply state: u201cThe authors received no specific funding for this work.u201d

<u>Reply:</u> The Financial Disclosure statement has been amended.

REVIEWER 1

Major issues

In regard to the protein expression then I noted from the sequence of the Pf P52-P36 fusion protein provided in Fig. S4 that, if this is the full sequence of the recombinant protein being expressed, then it appeared to lack an N-terminal secretory signal peptide. It is my understanding that if the protein is to be secreted by a eukaryotic (insect) cell then this would be required for directing the protein to the secretory pathway and therefore "correct" expression. I think that the authors are expecting the protein to be in the cell culture supernatant because they resolve supernatants and not cell pellets in Fig. S4C. Could this be a reason for the difficulties in trying to express this and the other proteins being tested? Perhaps I missed it but I was unable to get this information from the methods section. If this is the case then it should be highlighted in the paper (perhaps discussion) so that other researchers are not dissuaded from attempting expression for themselves. To be fully transparent, and to be helpful to the community, then the authors must provide the full protein sequence information of all the recombinant proteins they have tried to express.

Reply R1.1: We agree with the reviewer's comment. To avoid any confusion, we have provided more information on the employed constructs by generating an additional table in the 'Supporting Information' (**Table S1**) that contains a detailed overview of all constructs employed in this work. In addition, we have provided more details in the Materials and Methods section, which now reads as follows:

"The Bac-to-Bac Expression system (Thermo Fisher Scientific) was used according to the manufacturer's protocol to produce recombinant baculovirus. In brief, a pFastBac construct containing the PfP52-P36 gene preceded by an N-terminal GP64 signal peptide and TEV-protease cleavable 8xHis-tag was transformed into DH10Bac competent cells. All N-linked glycosylation sites were left intact."

Hence, a signal peptide was employed and we thus expect the fusion protein to be secreted into the cell culture's supernatant. The GP64 signal peptide is derived from baculoviruses and widely used in eukaryotic expression vectors to drive high-yield secretion of recombinant proteins. As the signal peptide is cleaved after secretion, we therefore opted to show only the sequence of the secreted protein in **Fig. S4**.

In my view the authors need to be more tempered with the interpretation of the EM and SAXS data (Fig.3). In several places in the manuscript they state that these data "experimentally validate" the head to tail model of the P52-P36 heterodimer. I would argue that the SAXS and the negative stain EM data could be open to other possible interpretations such as a head-to-head arrangement. The authors must provide higher resolution data to support their statement, or at least temper their language; for example, "consistent with" rather than "validate".

Reply R1.2: We fully agree with this comment and have adapted the text accordingly.

Minor issues

From the sequence provided in Fig. S4 it didn't appear that the authors mutated the N-linked glycosylation sites which is often done for proteins expressed in eukaryotic systems. This should be explicitly mentioned as a guide to others.

Reply R1.3: While the mutation of N-linked glycosylation sites can indeed be a successful strategy when it comes to recombinant protein production, these sites were left intact for the recombinant production of the PfP52-P36 fusion protein based on two arguments. First, the N-linked glycosylation sites of recombinantly produced malarial 6-Cys proteins are typically left intact when generating them in insect or mammalian cell lines (PMIDs: 26612952, 33480416, 35308105, 36153317). Second, recombinant production of any construct (single and fusion proteins) did not work in *E. coli* in our hands. This leads us to believe that retaining the glycans is important for proper folding of P36 and P52. In addition, we think they need each other to remain stable, which is why the fusion construct has been generated. As indicated in Reply R1.1, we now explicitly mention that the N-linked glycosylation sites were left intact.

As with point 2 above, the authors need to be more precise with their scientific language. It is an overinterpretation to say "Antibodies targeting B9 do not inhibit SPZ invasion" when the antibodies they are using are against an epitope tag inserted into either the N or C terminus of the protein. I'm sure the authors are aware of protective and non-protective epitopes and so their statement is too general and definitive given the data they provide.

Reply R1.4: We fully agree with this comment and have modified the text accordingly to make it clear that inhibition is mediated by anti-tag antibodies against engineered epitope tags, and we explicitly mention as a limitation that the epitope tagging strategy does not address whether native epitopes are immunogenic or protective.

In summary, publishing the negative data and structural modelling data in this paper is completely acceptable; however, given the experimental caveats I have mentioned, I would worry that it would influence the field to the extent that these vaccine candidates would not be explored further by other research groups. For publication, I would suggest that the authors temper some of their negative claims and be more forthcoming about the details of the protein expression constructs they have tested, highlighting any caveats.

Reply R1.5: By addressing all comments above, we believe to have provided a clear answer to this summarizing comment.

REVIEWER 2

Major issues

To improve their MS, it would be useful of the authors could provide (or display more prominently if I missed them) more details of exactly which constructs/sequences were used for each experiment. For example, Figure S1 suggests prediction data for residues approx. 31-301 while Fig 1 PAE plots show data for residue numbers 1-540 or so (presumably 1-270? For P36 31-303 and the remainder (271-??)? for P52). But then Figure 4B (sadly no numbering included in the cartoon) suggests the two protein subunits have rather different sizes, with regions outside D1 and D2 being of potential importance but not discussed – were these regions present in the various (His-tagged?) constructs used for prediction/production trials? Were they – as is indicated in the cartoon – present in the tagged constructs?

Reply R2.1: We agree with the reviewer's comment and have included an additional table in the 'Supporting Information' (**Table S1**) that contains a detailed overview of all constructs employed in this work.

Minor issues

The authors also might quickly check Alphafold3 predictions to ensure they corroborate the Alphafold-multimer derived ones. It might also be interesting (and would not take long) to predict the structure of their poly-Gly-linked heterodimer with a (theoretical) poly-Gly linked homodimer and to then predict what "happens" if they include a molecule of P36 or of P52 along with the Gly-linked homodimer in an AF3 prediction. If this displaces the homodimeric partner it would strengthen their case for the heterodimer predominating over the homodimer.

Reply R2.2: Following the reviewer's suggestion, we reran all hetero- and homodimer complexes using AlphaFold 3 through the AlphaFold server. The results have been appended to this rebuttal such that the reviewer may appreciate the outcome (**see below**). The complexes i) are predicted with very robust metrics (pLDDT, ipTM, and PAE) and ii) have quasi-identical architectures and interfaces. We have also looked into the second suggestion provided by the reviewer, by predicting the structures of linked P36-P36 and P52-P52 homodimers to which stand-alone P52 and P36 was "added", respectively. From the predicted structures, it is clear that the P36-P52 heterodimer is consistently formed. However, we have decided not to include the latter into the manuscript, as it would require biochemical evidence to convincingly prove that the heterodimer predominates over the homodimer. In addition (and as mentioned in the manuscript), we have no evidence that the homodimers may be relevant *in vivo*.

Just a small point – they should explain why they included data for an anti-TRAP antibody (ie what is TRAP?) in their report.

Reply R2.3: TRAP (Thrombospondin-Related Anonymous Protein) is a well characterized sporozoite microneme protein, and was used here as a microneme marker (in **Fig 4** and **new Fig S6**). This is now specified in the text.

REVIEWER 3

Major issues

1. How physiologically relevant is the epitope-tagging neutralization strategy to naturally occurring antibody responses?
The central conclusion is that P36 and P52 contain "vulnerable sites" accessible to neutralizing antibodies. However, the study relies entirely on artificial insertion of large epitope tags (3xFlag/V5) and high-affinity anti-tag monoclonal antibodies. It remains unclear whether endogenous epitopes in these regions are naturally immunogenic or structurally accessible in wild-type parasites. The authors should discuss more explicitly the limitations of extrapolating from artificial tags to native antibody recognition. Ideally, additional supporting evidence that would make for a stronger manuscript could include:
• structural surface accessibility analysis of native residues in these regions;
• conservation/immunogenicity analyses across species;
• testing polyclonal sera or peptide antibodies directed against native sequences.

Reply R3.1: Indeed, the epitope tagging neutralization approach shows that the P36-P52 complex is accessible to the action of neutralizing antibodies, but does not address whether P36/P52 contain immunogenic sequences nor that antibodies targeting native epitopes would inhibit infection. The same limitation stands for the experiments with B9. We have modified the text accordingly to make it clear that inhibition is mediated by anti-tag antibodies against engineered epitope tags, and we explicitly mention the limitation relative to native epitopes. Nevertheless, based on our data identifying domains in the P36-P52 heterodimer that are accessible to antibody neutralization, we believe that these antigens should be considered as potential new targets. We acknowledge that future studies will be required to determine whether native sequences are immunogenic and associated with protection against infection.

2. AlphaFold predictions vs. experimental data: You validated the head-to-tail architecture of the P. falciparum P36-P52 fusion protein using negative stain EM and SAXS. However, your functional epitope tagging and neutralization experiments were performed in P. berghei. Is it possible to try and express the P. berghei recombinant P36-P52 and then experimentally validate the P. berghei P36-P52 structure (e.g., by SAXS or EM) to confirm that the membrane-distal vs. -proximal domain orientations are identical between species? If not, how confident are you that domain accessibility is conserved?

Reply R3.2: We obviously attempted to recombinantly produce the *P. berghei* P52-P36 fusion protein, but unfortunately this construct did not express well in the insect cells. However, we are still convinced that the "head-to-tail" architectures are identical between species based on two arguments. First, other heterodimer complexes of 6-Cys proteins containing two domains adopt this orientation (PMIDs: 23511632, 35308105). Second, the AlphaFold predictions of both individual the proteins and their complexes presented in this paper are consistently robust and the predicted interaction interface appears to be strongly conserved as shown in **Figures 2** and **S1**.

3. Surface Accessibility Paradox: You state that neither P36 nor P52 could be detected on the sporozoite surface, even after activation. Yet, anti-tag antibodies clearly neutralize the

parasites. The inability to detect P36/P52 on the sporozoite surface is an important observation and deserves more experimental detail in the Results rather than only Discussion. If the proteins are not on the surface, how are the antibodies accessing them? Could neutralization be occurring intra-cellularly during the transient moment of microneme discharge, or is there a specific "point of no return" during invasion where these epitopes become briefly exposed?

Reply R3.3: As requested, we provide additional data (**new Fig S6**) to document that we could not detect P36 or P52 on the surface of activated sporozoites, using two different activation protocols (stimulation of microneme secretion by EtOH/BSA or in the presence of host cells). In both conditions TRAP was readily detected on the surface of non-permeabilized sporozoites (**new Fig S6A-B**). Importantly, P36 (but not P52) could be detected in the supernatant of activated sporozoites (**new Fig S6C**) confirming that it is exposed extracellularly. We also show that anti-Flag antibodies do not inhibit cell traversal of sporozoites expressing Flag-tagged P36 or P52 (**new Fig 4G**). Our results are in line with previous work showing that P36 and P52 were not detected on the sporozoite surface (refs 30, 45, 46). Altogether, our results suggest that P36-P52 are only exposed briefly at the parasite surface and/or in very small amounts. We have modified the text in the discussion as follows:

"*Our data show that anti-Flag antibodies inhibit infection of host cells by tagged parasites but have no effect on sporozoite cell traversal activity, consistent with the exclusive role of P36 and P52 during productive invasion [16,31,34,41]. It is possible that, following microneme secretion, P36 and P52 are exposed only briefly at the parasite surface at the time of host cell invasion, and/or in very small amounts, below the detection limit. Transient surface exposure would still allow high affinity antibodies (such as the anti-tag monoclonal antibodies used in this study) to bind to the P36-P52 complex and block its function.*"

Minor issues

1. Considering the low proteins yield and issues with purity (including insect cells contaminants) that the authors experienced, did they consider trying other protein expression platforms (e.g. mammalian HEK29, E. coli etc)

Reply R3.4: We thank the reviewer for this question. The (moderate) success with the *P. falciparum* P52-P36 fusion protein is the result of 6 years of hard (and frustrating) work by author Lien Boeykens. Lien has tried various expression systems to recombinantly produce the individual 6-Cys proteins at first: *E. coli* (various strains including SHuffle), HEK293T/F cells, *Leishmania tarentolae*, and Sf9/21 cells. Despite the optimization of various parameters, recombinant production consistently failed. Successes were finally obtained through the help of Wai-Hong Tham and Melanie Dietrich (who are acknowledged in the manuscript) for PfP52 and through the generation of the fusion protein, of which all results have been reported in this manuscript. Our interaction with the Tham lab has made it clear to us, that P36 and P52 are intrinsically recalcitrant proteins to work with. The team therefore applauds Lien's persistence in this project as she has dealt with countless failures over a long period of time before obtaining the results for PfP52 and the PfP52-P36 fusion protein.

2. The recombinant construct used in the SAXS/EM data to validate the architecture of the P36-52 heterodimer contains an artificial linker and contaminants and should be interpreted

with caution. The authors should comment on this as the Sf9-derived protease contamination (Cathepsin L) causes minor concerns about the structural integrity of the recombinant proteins used for SAXS and EM, which could subtly influence the resulting models.

Reply R3.5: We agree with this comment. It is for this reason that we have very openly reported the problems regarding recombinant production and purification in the manuscript. This resonates with the comment made by reviewer 1 and we have adapted the text accordingly to temper the interpretation of the EM and SAXS data (*cfr*. Reply R1.2).

3. In lines 387-390 the authors state that P. falciparum P36 and P52 are not functional/cannot be replaced in P. berghei but then in liens 403 – 405 they mention they went ahead and did exactly this to generate the triple falciparumized line PbPfP52P36B9. It would be good to clarify the discrepancy. And could the earlier comment explain why this PbPfP52P36B9 line showed very low levels of invasion?

Reply R3.6: We don't think there is any discrepancy here. Replacement of *P. berghei* P36-P52 by *P. falciparum* P36-P52 results in a KO-like phenotype. The same observation was made with B9. Therefore, we hypothesized that P36-P52 and B9 may assemble in a trimeric complex, which would explain why introduction of only P26-P52 or only B9 from P. falciparum would not be sufficient to maintain infectivity of transgenic *P. berghei* sporozoites. This is why we tested whether genetic complementation with the three proteins would be functional. We modified the text to clarify this point in the result and discussion sections, as follows:

"Previous inter-species genetic complementation studies in P. berghei showed that substitution of P. berghei P36 and P52 by P. yoelii homologous proteins maintained SPZ infectivity, while replacement by P. falciparum or P. vivax P36 and P52 led to non-infectious SPZs [106]. Similarly, P. berghei B9 could be replaced by P. yoelii but not by P. falciparum B9 propeller [107]. Given the similar phenotype of P36/P52 and B9 gene deletion mutants, we hypothesized that the three proteins might depend on each other as part of a functional tripartite complex required for SPZ invasion."

"Our data show that genetically engineered P. berghei SPZs carrying P. falciparum P36, P52 and B9 coding sequences are non-infective, strongly suggesting that additional factors are required for SPZ invasion of host cells. Alternatively, altered expression, trafficking or folding of P. falciparum proteins in P. berghei SPZs could also explain this phenotype. We also found that AlphaFold predictions of the P. falciparum, P. berghei, P. yoelii and P. vivax P36-P52-B9 complexes display very poor confidence scores. Hence, apart from the shared phenotype of gene deletion mutants, it remains unclear whether there is any functional or structural link between the P36-P52 heterodimer and B9. Further work is thus needed to elucidate the molecular function of these SPZ 6-Cys proteins."

4. Was the level of expression of the tagged versions (e.g., FlagN-P36 vs. P36-FlagC) quantified to ensure that differences in neutralization weren't simply due to different amounts of protein being present?

Reply R3.7: We verified that tagged proteins are expressed (Fig 4 and 5) and that genetically modified parasites show normal invasive capacity, demonstrating that the function of tagged proteins is preserved, but we did not quantified protein abundance.

5. The rationale for selecting HepG2 cells rather than primary hepatocytes or more physiologically relevant liver models should be briefly discussed. For example, would it be predicted these antibodies would lead to neutralisation in vivo.

Reply R3.8: We thank the reviewer for this comment. The HepG2 cell model is a robust cellular system to measure sporozoite invasion in culture conditions so we did not test other cell lines or primary hepatocytes. However, we agree that testing neutralization in vivo will be an important step. We already planned to adapt the tagging-neutralization strategy for in vivo testing in mice, which is not trivial as it requires extensive optimization (validation of antibody doses and bioavailability).

6. The last statements in the abstract and discussion slightly overstate the translational implications for vaccine development given the absence of native anti-P36/P52 antibody data.

Reply R3.9: based on our data identifying domains in the P36-P52 heterodimer that are accessible to antibody neutralization, we believe that these antigens should be considered as potential new targets. We modified the text to specify the limitation relative to native epitopes, and clearly refer to P36-P52 as potential targets. We acknowledge that future studies will be required to determine whether native sequences are immunogenic and associated with protection against infection.

7. The authors should clarify whether insertion of tags affected sporozoite fitness in mosquitoes, salivary gland invasion, or motility prior to hepatocyte infection.

Reply R3.10: Since P36, P52 and B9 are exclusively required for productive invasion of host cells, we verified that tagging does not affect host cell infection (**Fig 4D** and **5C**). We also tested for the P36-FLagC and FlagN-P52 lines that cell traversal activity is normal and not affected by anti-Flag antibodies (**new Fig 4G**). Unfortunately, we did not keep records of sporozoite numbers for all the experiments included in the manuscript. However, we did not observe any defect in mosquito infection, with numbers of salivary gland sporozoites in the usual range, as shown below:

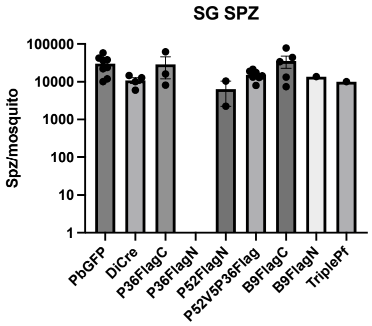

8. In the Results section (page 21, line 406), the line is called PbPfP52P36B9. In the Materials and Methods (page 32, line 66), it is called PbPfP52P36B9 but also referred to as

PbPfP36P52B9 in Supplementary Figure S7 (page 49, line 1098). Please standardize the name throughout the manuscript and supplement.

Reply R3.11: We have modified the text and now refer to this line as PbPfP52P36B9 throughout the manuscript.

ALPHAFOLD3 PREDICTIONS (*Related to reply R2.2)*

Heterodimers

PfP36-P52 heterodimer
ipTM = 0.85
pTM = 0.86

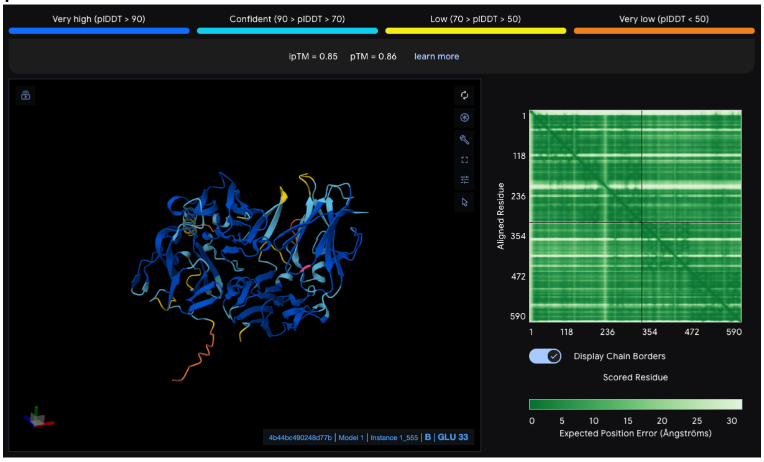

PbP36-P52 heterodimer
ipTM = 0.87
pTM = 0.88

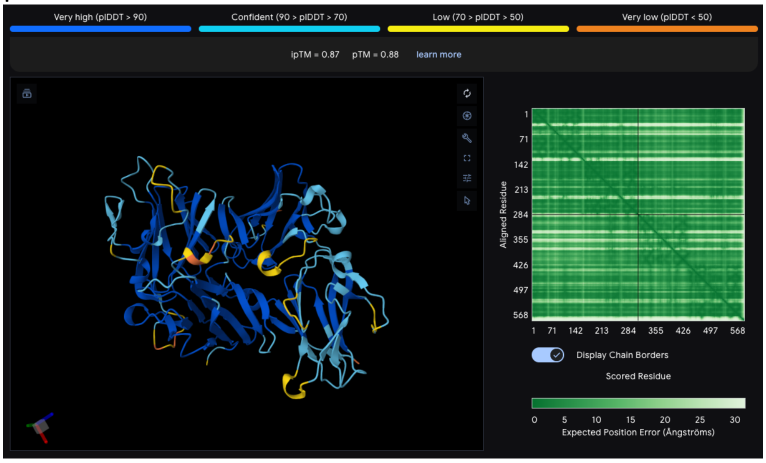

PvP36-P52 heterodimer
ipTM = 0.82
pTM = 0.88

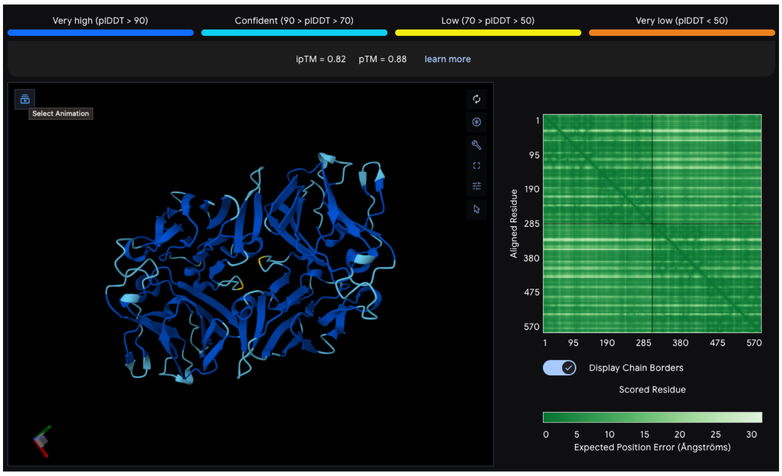

PyP36-P52 heterodimer
ipTM = 0.84
pTM = 0.86

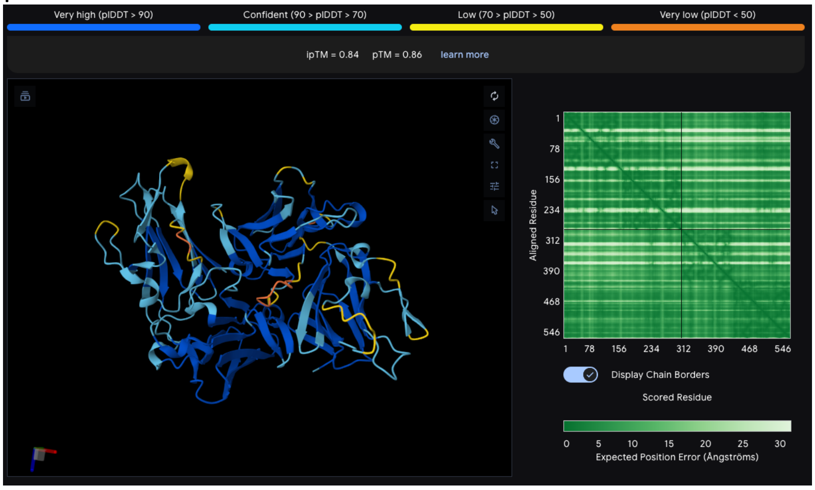

Homodimers

PfP36-P36 homodimer
ipTM = 0.65
pTM = 0.76

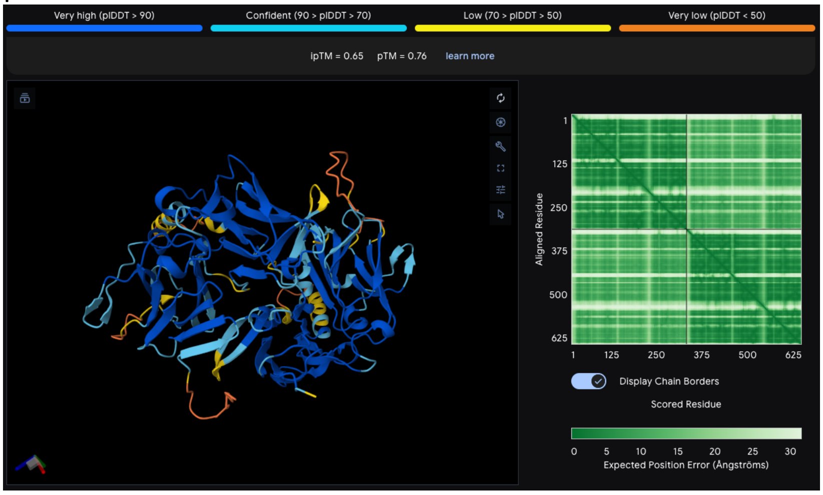

PfP52-P52 homodimer
ipTM = 0.79
pTM = 0.83

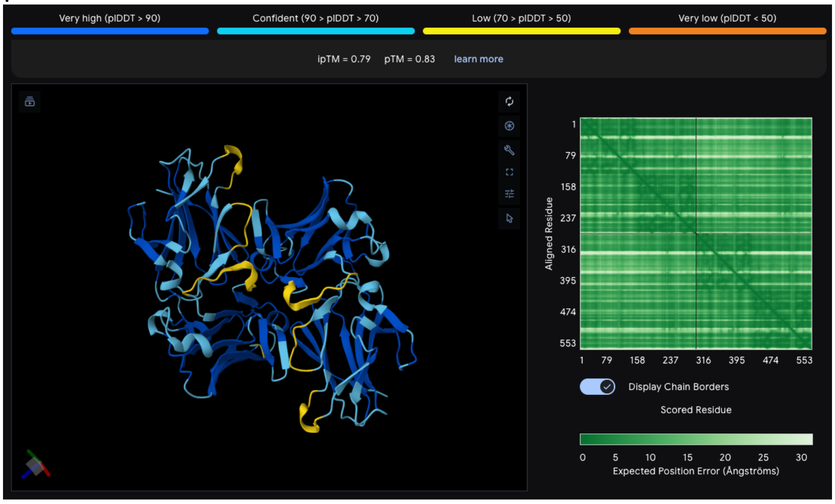

PbP36-P36 homodimer
ipTM = 0.81
pTM = 0.85

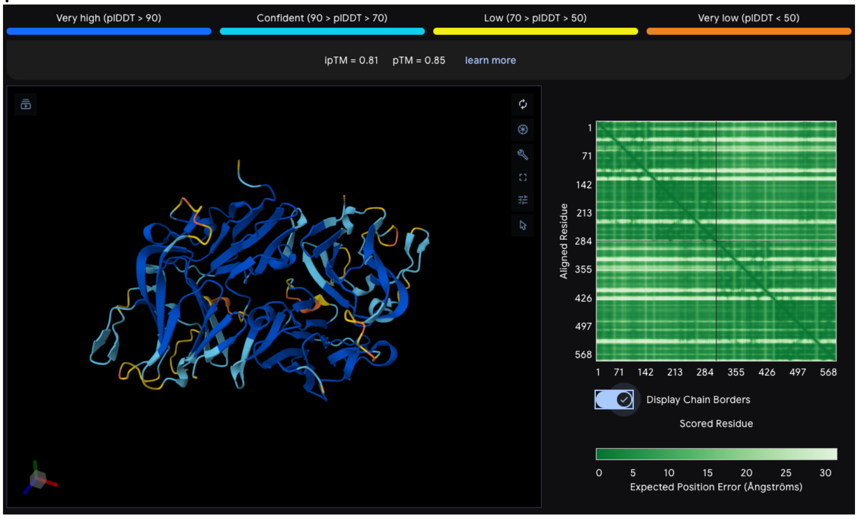

PbP52-P52 homodimer
ipTM = 0.83
pTM = 0.84

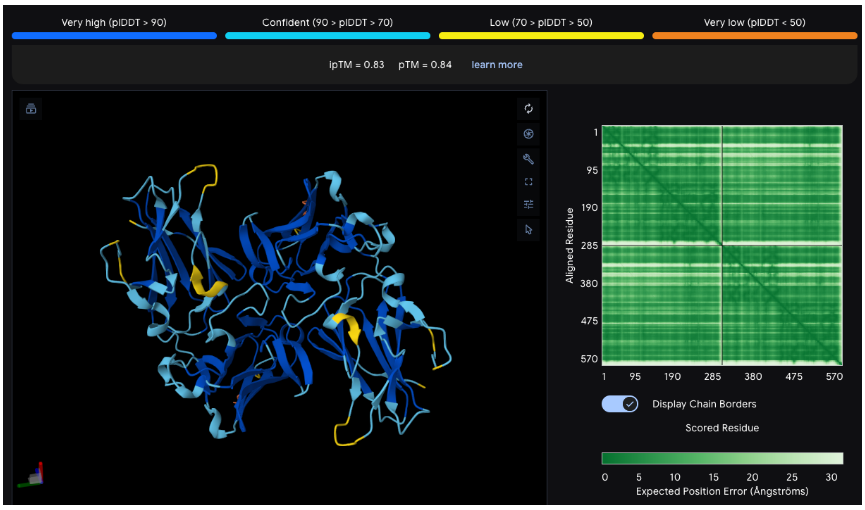

PvP36-P36 homodimer
ipTM = 0.83
pTM = 0.88

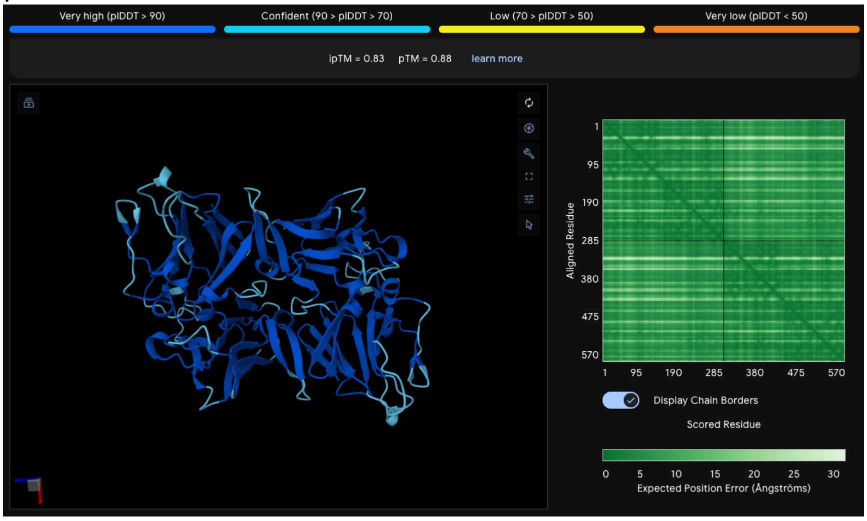

PvP52-P52 homodimer
ipTM = 0.78
pTM = 0.82

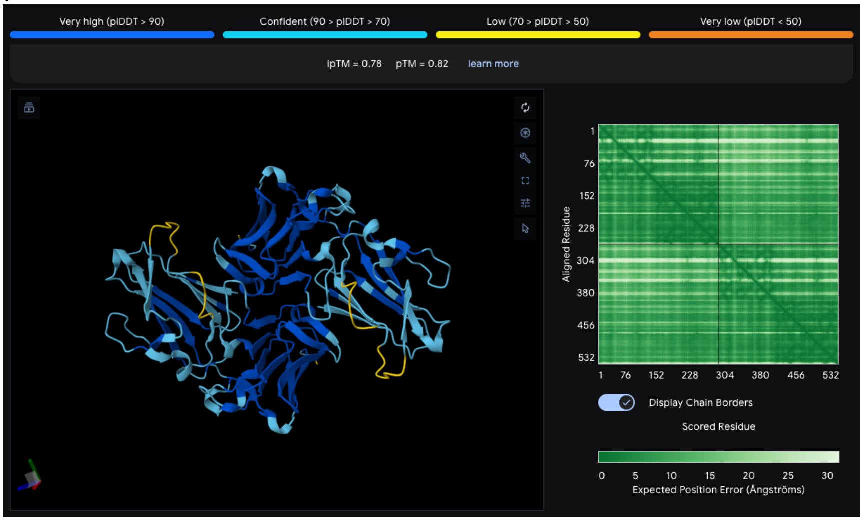

PyP36-P36 homodimer
ipTM = 0.82
pTM = 0.86

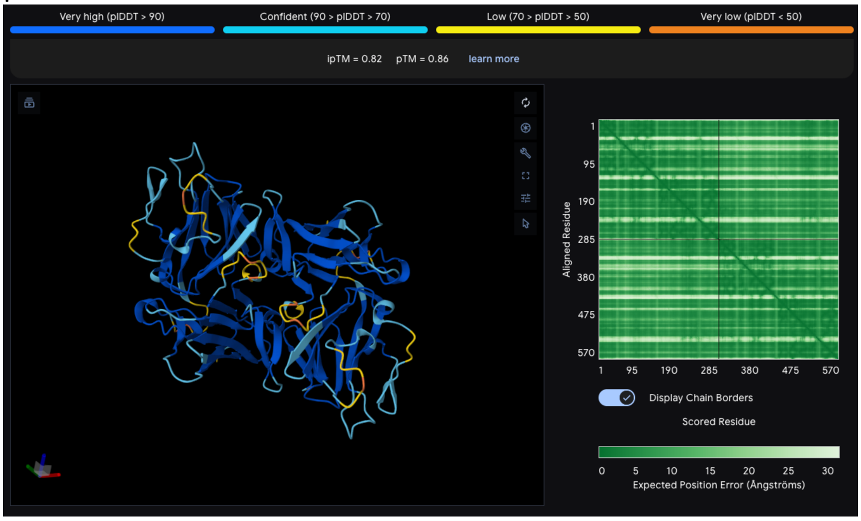

PyP52-P52 homodimer
ipTM = 0.46
pTM = 0.63

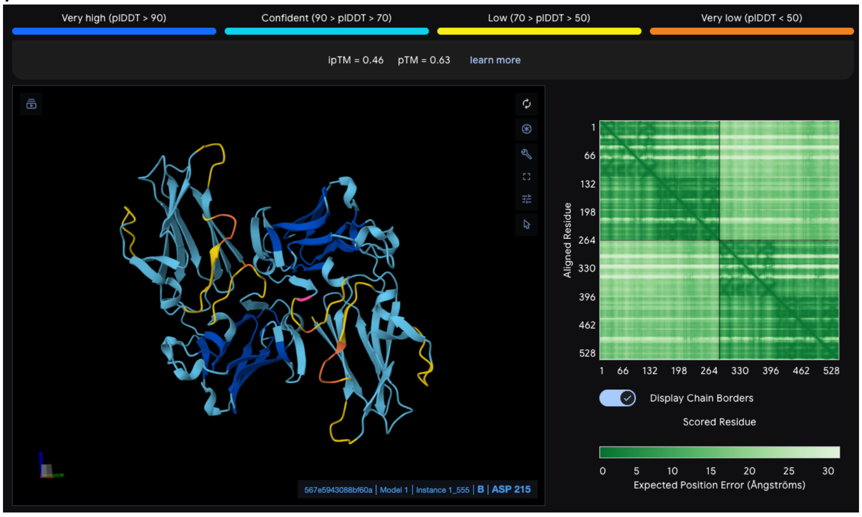

Fusion proteins

PfP52-P36 fusion
pTM = 0.72

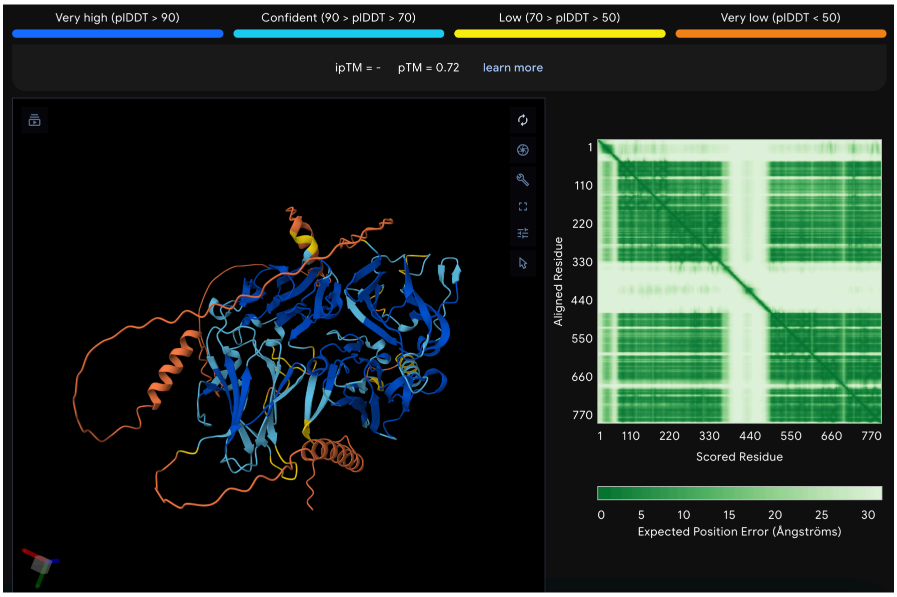

Fusion proteins with “extra partner”

PfP52-P52 fusion + PfP36 monomer
ipTM = 0.59
pTM = 0.46

P36 monomer breaks P52-P52 homodimer to form a heterodimer

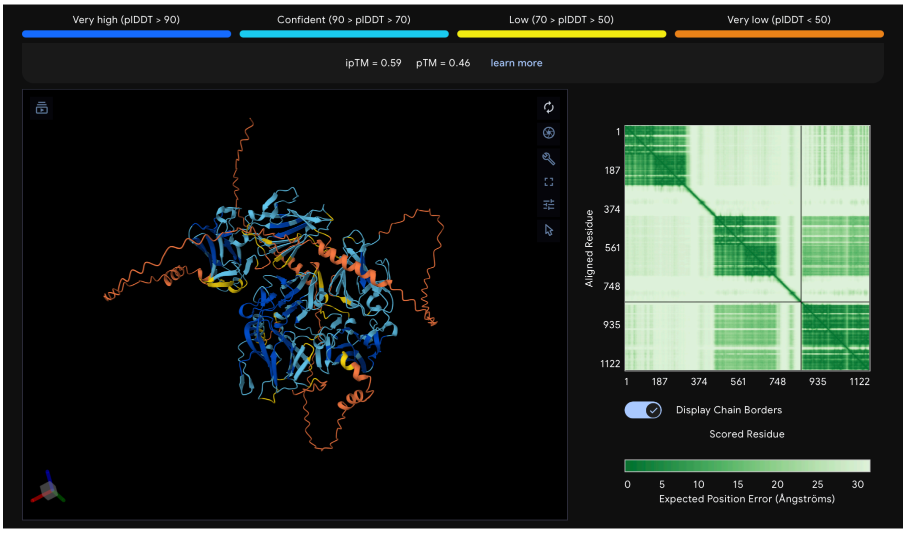

PfP36-P36 fusion + PfP52 monomer
ipTM = 0.60
pTM = 0.50

P52 monomer breaks P36-P36 homodimer to form a heterodimer

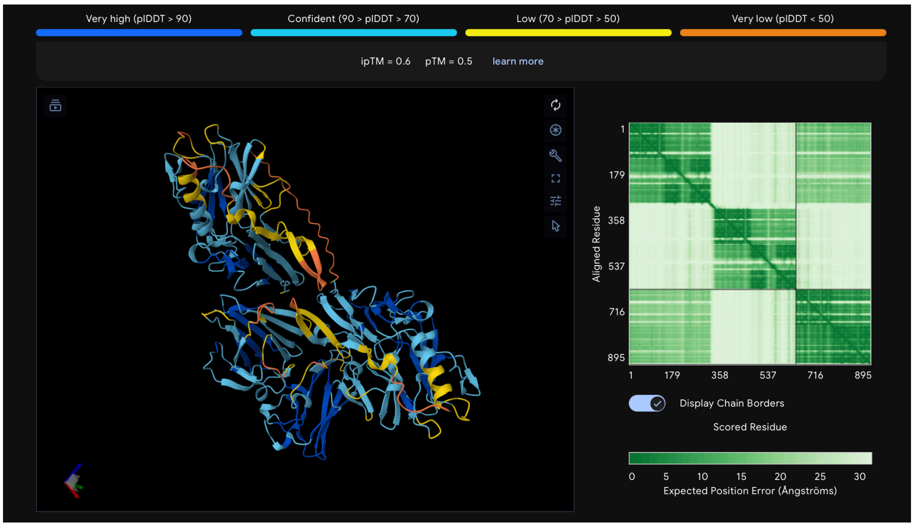
Very high (pIDDT > 90)
Confident (90 > pIDDT > 70)
Low (70 > pIDDT > 50)
Very low (pIDDT < 50)
ipTM = 0.6 pTM = 0.5 learn more
Aligned Residue
1
179
358
537
716
895
Display Chain Borders
Scored Residue
0 5 10 15 20 25 30
Expected Position Error (Ångströms)
