# Supplementary material for: A structure-based epitope tagging approach identifies vulnerable sites on the malarial P36-P52 protein complex for antibody-mediated neutralization of Plasmodium sporozoites
Source: PLoS Pathog. 2026 Jul 8;22(7):e1014418. doi: 10.1371/journal.ppat.1014418 (PMC13372241; doi:10.1371/journal.ppat.1014418)
Supplement: Peer review history

# Peer review: Decision Letter 0

**DOI:** 10.1371/journal.ppat.1014418.r001

**Authors:** Dominique Soldati-Favre, Daniel Fernandez-Ruiz

18 May 2026

PPATHOGENS-D-26-00836

A structure-based epitope tagging approach identifies vulnerable sites on the malarial P36-P52 protein complex for antibody-mediated neutralization of Plasmodium sporozoites

PLOS Pathogens

Dear Dr. Silvie,

Thank you for submitting your manuscript to PLOS Pathogens. After careful consideration, we feel that it has merit but does not fully meet PLOS Pathogens's publication criteria as it currently stands. Therefore, we invite you to submit a revised version of the manuscript that addresses the points raised during the review process.

Please submit your revised manuscript by Jul 17 2026 11:59PM. If you will need more time than this to complete your revisions, please reply to this message or contact the journal office at plospathogens@plos.org. When you're ready to submit your revision, log on to https://www.editorialmanager.com/ppathogens/ and select the 'Submissions Needing Revision' folder to locate your manuscript file.

Please include the following items when submitting your revised manuscript:

* A letter that responds to each point raised by the editor and reviewer(s). You should upload this letter as a separate file labeled 'Response to Reviewers'. This file does not need to include responses to any formatting updates and technical items listed in the 'Journal Requirements' section below.

* A marked-up copy of your manuscript that highlights changes made to the original version. You should upload this as a separate file labeled 'Revised Manuscript with Track Changes'.

* An unmarked version of your revised paper without tracked changes. You should upload this as a separate file labeled 'Manuscript'.

If you would like to make changes to your financial disclosure, competing interests statement, or data availability statement, please make these updates within the submission form at the time of resubmission. Guidelines for resubmitting your figure files are available below the reviewer comments at the end of this letter.

As the corresponding author, your ORCID iD is verified in the submission system and will appear in the published article. PLOS supports the use of ORCID, and we encourage all coauthors to register for an ORCID iD and use it as well. Please encourage your coauthors to verify their ORCID iD within the submission system before final acceptance, as unverified ORCID iDs will not appear in the published article. Only the individual author can complete the verification step; PLOS staff cannot verify ORCID iDs on behalf of authors.

We look forward to receiving your revised manuscript.

Kind regards,

Daniel Fernandez-Ruiz, PhD

Guest Editor

PLOS Pathogens

Dominique Soldati-Favre

Section Editor

PLOS Pathogens

Sumita Bhaduri-McIntosh

Editor-in-Chief

PLOS Pathogens

orcid.org/0000-0003-2946-9497

-->Michael Malim

Editor-in-Chief

PLOS Pathogens

orcid.org/0000-0002-7699-2064

**Additional Editor Comments:**

In addition to addressing the reviewer comments in full, please pay particular attention to the following points when revising the manuscript:

First, please moderate the interpretation of the anti-tag antibody experiments. The current data show that antibodies against engineered epitope tags can inhibit invasion, but they do not yet establish that native P36/P52 epitopes are naturally immunogenic or that antibodies against native P36/P52 sequences would neutralize infection. Claims relating to vulnerable epitopes, vaccine relevance, or therapeutic antibody targeting should therefore be revised accordingly unless additional supporting data are provided.

Second, please clarify and expand the description of all recombinant and parasite constructs used in the study, including the precise sequences, boundaries, tags, signal peptides, linkers, and relevant expression-design choices.

Third, please temper the language around the SAXS and negative-stain EM data. The reviewers were concerned that these data are more appropriately described as being consistent with the predicted P36-P52 architecture rather than as definitively validating it, unless stronger experimental evidence is provided.

Fourth, please address the apparent discrepancy between the reported difficulty detecting P36/P52 on the sporozoite surface and the ability of anti-tag antibodies to inhibit invasion. The manuscript would benefit from a clearer discussion, and where possible, additional data, explaining when and how these epitopes are likely to become antibody-accessible.

Finally, please substantially reconsider the interpretation of the triple PfP36/PfP52/PfB9 substitution experiment. As currently presented, the failure of these parasites to invade HepG2 cells is difficult to interpret as evidence against a functional P36/P52/B9 complex. This phenotype could have several explanations, including altered expression, folding, trafficking, localization, species-specific incompatibility, or dependence on additional P. falciparum factors. Please either provide additional evidence supporting the expression, localization, and appropriate trafficking of the substituted proteins, or reframe this result more cautiously as a negative observation that does not by itself exclude a functional P36/P52/B9 complex.

**Journal Requirements:**

If the reviewer comments include a recommendation to cite specific previously published works, please review and evaluate these publications to determine whether they are relevant and should be cited. There is no requirement to cite these works unless the editor has indicated otherwise.

1) Please ensure that the CRediT author contributions listed for every co-author are completed accurately and in full.

At this stage, the following Authors/Authors require contributions: Samhita Das, Lien Boeykens, Manon Loubens, Carine Marinach, Sylvie Briquet, Line De Vocht, Isabel Pintelon, Jean-Pierre Timmermans, Yann Sterckx, and Olivier Silvie. Please ensure that the full contributions of each author are acknowledged in the "Add/Edit/Remove Authors" section of our submission form.

The list of CRediT author contributions may be found here: https://journals.plos.org/plospathogens/s/authorship#loc-author-contributions

2) We ask that a manuscript source file is provided at Revision. Please upload your manuscript file as a .doc, .docx, .rtf or .tex. If you are providing a .tex file, please upload it under the item type u2018LaTeX Source Fileu2019 and leave your .pdf version as the item type u2018Manuscriptu2019.

3) Please provide an Author Summary. This should appear in your manuscript between the Abstract (if applicable) and the Introduction, and should be 150-200 words long. The aim should be to make your findings accessible to a wide audience that includes both scientists and non-scientists. Sample summaries can be found on our website under Submission Guidelines:

https://journals.plos.org/plospathogens/s/submission-guidelines#loc-parts-of-a-submission

4) We noticed that you used the phrase 'data not shown' in the manuscript. We do not allow these references, as the PLOS data access policy requires that all data be either published with the manuscript or made available in a publicly accessible database. Please amend the supplementary material to include the referenced data or remove the references.

5) We do not publish any copyright or trademark symbols that usually accompany proprietary names, eg ©,  ®, or TM  (e.g. next to drug or reagent names). Therefore please remove all instances of trademark/copyright symbols throughout the text, including:

- TM on pages: 24, and 30.

6) Please upload all main figures as separate Figure files in .tif or .eps format. For more information about how to convert and format your figure files please see our guidelines:

https://journals.plos.org/plospathogens/s/figures

7) We notice that your supplementary Figures are included in the manuscript file. Please remove them and upload them with the file type 'Supporting Information'. Please ensure that each Supporting Information file has a legend listed in the manuscript after the references list.

8) Some material included in your submission may be copyrighted. According to PLOS’s copyright policy, authors who use figures or other material (e.g., graphics, clipart, maps) from another author or copyright holder must demonstrate or obtain permission to publish this material under the Creative Commons Attribution 4.0 International (CC BY 4.0) License used by PLOS journals. Please closely review the details of PLOS’s copyright requirements here: PLOS Licenses and Copyright. If you need to request permissions from a copyright holder, you may use PLOS's Copyright Content Permission form.

Please respond directly to this email and provide any known details concerning your material's license terms and permissions required for reuse, even if you have not yet obtained copyright permissions or are unsure of your material's copyright compatibility. Once you have responded and addressed all other outstanding technical requirements, you may resubmit your manuscript within Editorial Manager.

Potential Copyright Issues:

i) Figure 4A. Please confirm whether you drew the images / clip-art within the figure panels by hand. If you did not draw the images, please provide (a) a link to the source of the images or icons and their license / terms of use; or (b) written permission from the copyright holder to publish the images or icons under our CC BY 4.0 license. Alternatively, you may replace the images with open source alternatives. See these open source resources you may use to replace images / clip-art:

- https://commons.wikimedia.org

- https://openclipart.org/.

ii) The following Figures contain screenshots: S4, and S7A. We are not permitted to publish these under our CC-BY 4.0 license, websites are usually intellectual property and are copyrighted.This includes peripheral graphics of the web browser such as icons and button. We ask that you please remove or replace it.

9) Please amend your detailed Financial Disclosure statement. This is published with the article. It must therefore be completed in full sentences and contain the exact wording you wish to be published.

1) State what role the funders took in the study. If the funders had no role in your study, please state: "The funders had no role in study design, data collection and analysis, decision to publish, or preparation of the manuscript."

2) If any authors received a salary from any of your funders, please state which authors and which funders..

If you did not receive any funding for this study, please simply state: u201cThe authors received no specific funding for this work.u201d

**Reviewers' Comments:**

Reviewer's Responses to Questions

**Part I - Summary**

Please use this section to discuss strengths/weaknesses of study, novelty/significance, general execution and scholarship.

Reviewer #1: Das et al. A structure-based epitope tagging approach identifies vulnerable sites on the malarial P36-P52 protein complex for antibody-mediated neutralization of Plasmodium sporozoites

This paper investigates the structure of several members of the 6-cys family of proteins (P36, P52 and B9) in Plasmodium spp. parasites and their role in the invasion of cells by the liver (sporozoite) stage of the parasite. The background to this study is that genetic studies in P. falciparum, P. berghei and P. yoelii have shown that P36, P52 and B9 are essential for productive invasion of hepatocytes, making them potential pre-erythrocytic vaccine targets. P36 is predicted to be secreted and therefore form a complex with the P52 which is predicted to be anchored to membrane with a GPI-anchor; the B9 protein is also predicted to be GPI-anchored. The authors provide structural modelling of the suggested complex of P36/P52 complex in different Plasmodium species and the interaction interface (Figs 1&2), and some structural support for the orientation of the complex using a P36-P52 fusion protein (Fig. 3). By genetically introducing epitope tags at both N and C-terminii in the P36, P52 and B9 proteins, they show evidence for inhibition of hepatocyte infection for C-terminal (but not N-terminal) tagged P36 and N-terminal (but not C-terminal) tagged P52 (Fig. 4); antibodies targeting epitopes tags at the N or C terminus of B9 had no effect on infection (Fig. 5). Finally, sequential replacement of p36, p52 and b9 with the P. falciparum orthologues in P. berghei resulted in sporozoites that were unable to infect hepatocytes (second half of Fig. 5).

The paper is very well written and the authors have done a very nice job on presenting the data making it easy to read. I sympathize with the authors with their documented struggles with protein expression. I'm sure the authors are fully aware that the vast majority of the structural work are structural predictions, which, as well as it is done here, tempers enthusiasm for the paper somewhat.

Reviewer #2: This clearly written MS describes an early exploration of a heterodimeric cysteine-rich sporozoite surface protein as a target for antibodies and hence for potential incorporation into a future vaccine. It describes a multidisciplinary project that spans structure prediction to help design constructs, the production of epitope-tagged versions easily targeted by available antibodies and the creation of parasite models to test the efficacy of this strategy. The results are very encouraging and provide a foundation for further study. It is of course hard to overstate the importance of developing more/better vaccines in this field.

The work appears to have been carried out carefully and to be technically sound. Production of the recombinant proteins in insect cells (an obvious choice of host) proved highly challenging, and even a construct in which heterodimer-forming P32 and P56 were joined by a flexible poly-glycine linker produced low yields of soluble protein. The authors could have explored other production hosts (P. pastoris for example) or tried different constructs (see below), but with no guarantee of success, so they persevered. Like the production struggles, the structural studies left many unanswered questions (eg regarding homodimerization versus heterodimerisation and even trimerization) but served their purpose.

In the absence of an easy route to raising antibodies against these challenging-to-produce proteins they sensible chose an epitope-tagging strategy. This proved informative with respect to providing a proof of concept, and offering clues about which region of the GPI-anchored heterodimer might provide the optimal epitope. Their inclusion of a third 6-diS protein, B9, in their study provided a reassuring negative control. The authors access to knockout strains and ability to complement these with tagged proteins adds strong value to the study.

Reviewer #3: Thank you for the opportunity to review the manuscript by Samhita Das and colleagues titled “A structure-based epitope tagging approach identified vulnerable site on the malarial P36-P52 protein complex for antibody-mediated neutralization of Plasmodium sporozoites”. In this manuscript the authors set out to understand whether other sporozoite proteins other than the major circumsporozoite protein (CSP) are targets of antibody-mediated neutralization. Specifically, they looked at the three 6-cysteine domain proteins P36, P52 and B9. Using a combination of AlphaFold-based structural prediction, experimental structural validation (negative stain EM and SAXS), and a novel epitope tagging strategy to map vulnerable sites on the P36-P52 heterodimer they show that:

1. The P36-P52 complex adopts a head-to-tail architecture with membrane-distal and membrane-proximal domains, conserved across multiple Plasmodium species.

2. Antibodies targeting membrane-distal domains (C-terminus of P36, N-terminus of P52) block sporozoite invasion in vitro, while antibodies targeting membrane-proximal domains do not.

3. B9, another 6-Cys protein essential for invasion, is not neutralized by antibodies targeting its N- or C-terminus.

4. There is no functional evidence for a tripartite P36-P52-B9 complex.

**********

**Part II – Major Issues: Key Experiments Required for Acceptance**

Please use this section to detail the key new experiments or modifications of existing experiments that should be absolutely required to validate study conclusions.

Generally, there should be no more than 3 such required experiments or major modifications for a "Major Revision" recommendation. If more than 3 experiments are necessary to validate the study conclusions, then you are encouraged to recommend "Reject".

Reviewer #1: I have the following points which I hope will be helpful:

In regard to the protein expression then I noted from the sequence of the Pf P52-P36 fusion protein provided in Fig. S4 that, if this is the full sequence of the recombinant protein being expressed, then it appeared to lack an N-terminal secretory signal peptide. It is my understanding that if the protein is to be secreted by a eukaryotic (insect) cell then this would be required for directing the protein to the secretory pathway and therefore “correct” expression. I think that the authors are expecting the protein to be in the cell culture supernatant because they resolve supernatants and not cell pellets in Fig. S4C. Could this be a reason for the difficulties in trying to express this and the other proteins being tested? Perhaps I missed it but I was unable to get this information from the methods section. If this is the case then it should be highlighted in the paper (perhaps discussion) so that other researchers are not dissuaded from attempting expression for themselves. To be fully transparent, and to be helpful to the community, then the authors must provide the full protein sequence information of all the recombinant proteins they have tried to express.

In my view the authors need to be more tempered with the interpretation of the EM and SAXS data (Fig.3). In several places in the manuscript they state that these data "experimentally validate” the head to tail model of the P52-P36 heterodimer. I would argue that the SAXS and the negative stain EM data could be open to other possible interpretations such as a head-to-head arrangement. The authors must provide higher resolution data to support their statement, or at least temper their language; for example, “consistent with” rather than “validate”.

Reviewer #2: To improve their MS, it would be useful of the authors could provide (or display more prominently if I missed them) more details of exactly which constructs/sequences were used for each experiment. For example, Figure S1 suggests prediction data for residues approx. 31-301 while Fig 1 PAE plots show data for residue numbers 1-540 or so (presumably 1-270? For P36 31-303 and the remainder(271-??)? for P52). But then Figure 4B (sadly no numbering included in the cartoon) suggests the two protein subunits have rather different sizes, with regions outside D1 and D2 being of potential importance but not discussed – were these regions present in the various (His-tagged?) constructs used for prediction/production trials? Were they – as is indicated in the cartoon – present in the tagged constructs?

Reviewer #3: Major comments:

1. How physiologically relevant is the epitope-tagging neutralization strategy to naturally occurring antibody responses?

The central conclusion is that P36 and P52 contain “vulnerable sites” accessible to neutralizing antibodies. However, the study relies entirely on artificial insertion of large epitope tags (3xFlag/V5) and high-affinity anti-tag monoclonal antibodies. It remains unclear whether endogenous epitopes in these regions are naturally immunogenic or structurally accessible in wild-type parasites. The authors should discuss more explicitly the limitations of extrapolating from artificial tags to native antibody recognition. Ideally, additional supporting evidence that would make for a stronger manuscript could include:

• structural surface accessibility analysis of native residues in these regions;

• conservation/immunogenicity analyses across species;

• testing polyclonal sera or peptide antibodies directed against native sequences.

2. AlphaFold predictions vs. experimental data: You validated the head-to-tail architecture of the P. falciparum P36-P52 fusion protein using negative stain EM and SAXS. However, your functional epitope tagging and neutralization experiments were performed in P. berghei. Is it possible to try and express the P. berghei recombinant P36-P52 and then experimentally validate the P. berghei P36-P52 structure (e.g., by SAXS or EM) to confirm that the membrane-distal vs. -proximal domain orientations are identical between species? If not, how confident are you that domain accessibility is conserved?

3. Surface Accessibility Paradox: You state that neither P36 nor P52 could be detected on the sporozoite surface, even after activation. Yet, anti-tag antibodies clearly neutralize the parasites. The inability to detect P36/P52 on the sporozoite surface is an important observation and deserves more experimental detail in the Results rather than only Discussion. If the proteins are not on the surface, how are the antibodies accessing them? Could neutralization be occurring intra-cellularly during the transient moment of microneme discharge, or is there a specific "point of no return" during invasion where these epitopes become briefly exposed?

**********

**Part III – Minor Issues: Editorial and Data Presentation Modifications**

Please use this section for editorial suggestions as well as relatively minor modifications of existing data that would enhance clarity.

Reviewer #1: Minor points:

From the sequence provided in Fig. S4 it didn’t appear that the authors mutated the N-linked glycosylation sites which is often done for proteins expressed in eukaryotic systems. This should be explicitly mentioned as a guide to others.

As with point 2 above, the authors need to be more precise with their scientific language. It is an overinterpretation to say “Antibodies targeting B9 do not inhibit SPZ invasion” when the antibodies they are using are against an epitope tag inserted into either the N or C terminus of the protein. I’m sure the authors are aware of protective and non-protective epitopes and so their statement is too general and definitive given the data they provide.

In summary, publishing the negative data and structural modelling data in this paper is completely acceptable; however, given the experimental caveats I have mentioned, I would worry that it would influence the field to the extent that these vaccine candidates would not be explored further by other research groups. For publication, I would suggest that the authors temper some of their negative claims and be more forthcoming about the details of the protein expression constructs they have tested, highlighting any caveats.

Reviewer #2: The authors also might quickly check Alphafold3 predictions to ensure they corroborate the Alphafold-multimer derived ones. It might also be interesting (and would not take long) to predict the structure of their poly-Gly-linked heterodimer with a (theoretical) poly-Gly linked homodimer and to then predict what “happens” if they include a molecule of P32 or of P56 along with the Gly-linked homodimer in an AF3 prediction. If this displaces the homodimeric partner it would strengthen their case for the heterodimer predominating over the homodimer.

Just a small point – they should explain why they included data for an anti-TRAP antibody (ie what is TRAP?) in their report.

Reviewer #3: Minor comments

1. Considering the low proteins yield and issues with purity (including insect cells contaminants) that the authors experienced, did they consider trying other protein expression platforms (e.g. mammalian HEK29, E. coli etc)

2. The recombinant construct used in the SAXS/EM data to validate the architecture of the P36-52 heterodimer contains an artificial linker and contaminants and should be interpreted with caution. The authors should comment on this as the Sf9-derived protease contamination (Cathepsin L) causes minor concerns about the structural integrity of the recombinant proteins used for SAXS and EM, which could subtly influence the resulting models.

3. In lines 387-390 the authors state that P. falciparum P36 and P52 are not functional/cannot be replaced in P. berghei but then in liens 403 – 405 they mention they went ahead and did exactly this to generate the triple falciparumized line PbPfP52P36B9. It would be good to clarify the discrepancy. And could the earlier comment explain why this PbPfP52P36B9 line showed very low levels of invasion?

4. Was the level of expression of the tagged versions (e.g., FlagN-P36 vs. P36-FlagC) quantified to ensure that differences in neutralization weren't simply due to different amounts of protein being present?

5. The rationale for selecting HepG2 cells rather than primary hepatocytes or more physiologically relevant liver models should be briefly discussed. For example, would it be predicted these antibodies would lead to neutralisation in vivo.

6. The last statements in the abstract and discussion slightly overstate the translational implications for vaccine development given the absence of native anti-P36/P52 antibody data.

7. The authors should clarify whether insertion of tags affected sporozoite fitness in mosquitoes, salivary gland invasion, or motility prior to hepatocyte infection.

8. In the Results section (page 21, line 406), the line is called PbPfP52P36B9. In the Materials and Methods (page 32, line 66), it is called PbPfP52P36B9 but also referred to as PbPfP36P52B9 in Supplementary Figure S7 (page 49, line 1098). Please standardize the name throughout the manuscript and supplement.

**********

PLOS authors have the option to publish the peer review history of their article (what does this mean?). If published, this will include your full peer review and any attached files.

If you choose “no”, your identity will remain anonymous but your review may still be made public.

**Do you want your identity to be public for this peer review?**  For information about this choice, including consent withdrawal, please see our Privacy Policy.

Reviewer #1: No

Reviewer #2: **Yes:**  Paul Barlow

Reviewer #3: No

**Figure resubmission:**

-->While revising your submission, we strongly recommend that you use PLOS’s NAAS tool (https://ngplosjournals.pagemajik.ai/artanalysis) to test your figure files. NAAS can convert your figure files to the TIFF file type and meet basic requirements (such as print size, resolution), or provide you with a report on issues that do not meet our requirements and that NAAS cannot fix.

After uploading your figures to PLOS’s NAAS tool - https://ngplosjournals.pagemajik.ai/artanalysis, NAAS will process the files provided and display the results in the "Uploaded Files" section of the page as the processing is complete. If the uploaded figures meet our requirements (or NAAS is able to fix the files to meet our requirements), the figure will be marked as "fixed" above. If NAAS is unable to fix the files, a red "failed" label will appear above. When NAAS has confirmed that the figure files meet our requirements, please download the file via the download option, and include these NAAS processed figure files when submitting your revised manuscript.

**Reproducibility:**

To enhance the reproducibility of your results, we recommend that authors of applicable studies deposit laboratory protocols in protocols.io, where a protocol can be assigned its own identifier (DOI) such that it can be cited independently in the future. Additionally, PLOS ONE offers an option to publish peer-reviewed clinical study protocols. Read more information on sharing protocols at https://plos.org/protocols?utm_medium=editorial-email&utm_source=authorletters&utm_campaign=protocols

# Author comment: Author response to Decision Letter 1

**DOI:** 10.1371/journal.ppat.1014418.r002

18 Jun 2026

Attachment Submitted filename: Das Boeykens et al_Response_to_reviewers.pdf

# Editor report: Decision Letter 1

**DOI:** 10.1371/journal.ppat.1014418.r003

**Authors:** Dominique Soldati-Favre, Daniel Fernandez-Ruiz, Dominique Soldati-Favre, Daniel Fernandez-Ruiz

25 Jun 2026

Dear Dr Silvie,

We are pleased to inform you that your manuscript 'A structure-based epitope tagging approach identifies vulnerable sites on the malarial P36-P52 protein complex for antibody-mediated neutralization of Plasmodium sporozoites' has been provisionally accepted for publication in PLOS Pathogens.

Before your manuscript can be formally accepted you will need to complete some formatting changes, which you will receive in a follow up email. A member of our team will be in touch with a set of requests.

Please note that your manuscript will not be scheduled for publication until you have made the required changes, so a swift response is appreciated.

IMPORTANT: The editorial review process is now complete. PLOS will only permit corrections to spelling, formatting or significant scientific errors from this point onwards. Requests for major changes, or any which affect the scientific understanding of your work, will cause delays to the publication date of your manuscript.

Should you, your institution's press office or the journal office choose to press release your paper, you will automatically be opted out of early publication. We ask that you notify us now if you or your institution is planning to press release the article. All press must be co-ordinated with PLOS.

Thank you again for supporting Open Access publishing; we are looking forward to publishing your work in PLOS Pathogens.

Best regards,

Daniel Fernandez-Ruiz, PhD

Guest Editor

PLOS Pathogens

Dominique Soldati-Favre

Section Editor

PLOS Pathogens

Sumita Bhaduri-McIntosh

Editor-in-Chief

PLOS Pathogens

orcid.org/0000-0003-2946-9497

Michael Malim

Editor-in-Chief

PLOS Pathogens

orcid.org/0000-0002-7699-2064

***********************************************************

Reviewer Comments (if any, and for reference):

# Editor report: Acceptance letter

**DOI:** 10.1371/journal.ppat.1014418.r004

**Authors:** Dominique Soldati-Favre, Daniel Fernandez-Ruiz, Dominique Soldati-Favre, Daniel Fernandez-Ruiz

Dear Dr Silvie,

We are delighted to inform you that your manuscript, "A structure-based epitope tagging approach identifies vulnerable sites on the malarial P36-P52 protein complex for antibody-mediated neutralization of Plasmodium sporozoites," has been formally accepted for publication in PLOS Pathogens.

We have now passed your article onto the PLOS Production Department who will complete the rest of the pre-publication process. All authors will receive a confirmation email upon publication.

The corresponding author will soon be receiving a typeset proof for review, to ensure errors have not been introduced during production. Please review the PDF proof of your manuscript carefully, as this is the last chance to correct any scientific or type-setting errors. Please note that major changes, or those which affect the scientific understanding of the work, will likely cause delays to the publication date of your manuscript. Note: Proofs for Front Matter articles (Pearls, Reviews, Opinions, etc...) are generated on a different schedule and may not be made available as quickly.

Soon after your final files are uploaded, the early version of your manuscript, if you opted to have an early version of your article, will be published online. The date of the early version will be your article's publication date. The final article will be published to the same URL, and all versions of the paper will be accessible to readers.

For Research Articles, you will receive an invoice from PLOS for your publication fee after your manuscript has reached the completed accept phase. If you receive an email requesting payment before acceptance or for any other service, this may be a phishing scheme. Learn how to identify phishing emails and protect your accounts at https://explore.plos.org/phishing.

Thank you again for supporting open-access publishing; we are looking forward to publishing your work in PLOS Pathogens.

Best regards,

Sumita Bhaduri-McIntosh

Editor-in-Chief

PLOS Pathogens

orcid.org/0000-0003-2946-9497

Michael Malim

Editor-in-Chief

PLOS Pathogens

orcid.org/0000-0002-7699-2064
